# Functional Role of YnfA, an Efflux Transporter in Resistance to Antimicrobial Agents in Shigella flexneri

**DOI:** 10.1128/aac.00293-22

**Published:** 2022-06-21

**Authors:** Tanuka Sen, Naresh K. Verma

**Affiliations:** a Division of Biomedical Science and Biochemistry, Research School of Biology, The Australian National University, Canberra, Australia

**Keywords:** *Shigella flexneri*, antimicrobial resistance, bacterial efflux pumps, YnfA, SMR transporters, EmrE

## Abstract

Shigella flexneri has become a significant public health concern accounting for the majority of shigellosis cases worldwide. Even though a multitude of efforts is being made into the development of a vaccine to prevent infections, the absence of a licensed global vaccine compels us to enormously depend on antibiotics as the major treatment option. The extensive-unregulated use of antibiotics for treatment along with natural selection in bacteria has led to the rising of multidrug-resistance *Shigella* strains. Out of the various mechanisms employed by bacteria to gain resistance, efflux transporters are considered to be one of the principal contributors to antimicrobial resistance. The small multidrug-resistance family consists of unique small proteins that act as efflux pumps and are involved in extruding various antimicrobial compounds. The present study aims to demonstrate the role of an efflux transporter YnfA belonging to the SMR family and its functional involvement in promoting antimicrobial resistance in S. flexneri. Employing various genetic, computational, and biochemical techniques, we show how disrupting the YnfA transporter, renders the mutant *Shigella* strain more susceptible to some antimicrobial compounds tested in this study, and significantly affects the overall transport activity of the bacteria against ethidium bromide and acriflavine when compared with the wild-type *Shigella* strain. We also assessed how mutating some of the conserved amino acid residues of YnfA alters the resistance profile and efflux activity of the mutant YnfA transporter. This study provides a functional understanding of an uncharacterized SMR transporter YnfA of *Shigella*.

## INTRODUCTION

*Shigella* species are a huge threat to public health, as they have a low infective dose in order of 10 to 100 organisms and is known to be the second most collective cause of diarrheal deaths worldwide (1–4). The absence of an effective and cross-protective vaccine against *Shigella* serotypes compels us to be dependent on the limited treatment options available (5–7). Employing the use of antibiotics in shigellosis cases is one of the key clinical treatment approaches which helps in decreasing the severity and extent of infection, the transmission of bacteria, and reduces the risk of further complications associated with *Shigella* infections (8–11). However, over the previous decades, there is an alarming increase of antimicrobial resistance (AMR) in various *Shigella* species and serotypes (12, 13). Shigellosis caused by AMR strains can increase the overall health care cost and escalate the chances of a poor outcome or death in infected individuals (12–14). The development of resistance in *Shigella* spp. is believed to be partly due to the process of natural selection, but the widespread and unregulated usage of antibiotics, has only amplified this problem (9, 12–17). The global patterns of antimicrobial resistance seen in *Shigella* strains differ with time and depends heavily on the geographical location and usage of particular antibiotics (9, 12–17). The genes contributing to AMR in *Shigella* are mostly plasmid-borne but some are even present on the chromosome (17). Multidrug resistance (MDR) in bacteria, can be due to numerous mechanisms such as the expulsion of antimicrobial compounds by efflux pumps, alteration of membrane permeability, mutational modification of the drug target, and lastly the expression of enzymes that can modify or inactivate the drug (18).

Gram-negative bacteria commonly utilize various efflux mechanisms, achieved by their membrane proteins which actively extrude the antimicrobial drugs out of the cell (19–22). This kind of active transport carried out by bacterial efflux pumps (EP) has been recognized as a major player in promoting antimicrobial resistance (19–22). Antimicrobials can function as important inducers, regulating expression or even overexpression of EP, and play a central role in bacteria in terms of its acquired and intrinsic drug resistance (22, 23). These EPs are mostly composed of one or more protein constituents and traverse through the entire cell membrane, binding and pumping out an extensive variety of drugs (23). Bacterial EPs can either be located in the inner membrane (IM) as a single-component transporter or form tripartite complexes consisting of an outer membrane protein, an IM protein along with a membrane fusion protein traversing through both the membranes (21–23). Six crucial families of efflux transporters are known: the multidrug and toxic compound extrusion (MATE) family, the small multidrug-resistance (SMR) family, the ATP binding cassette (ABC) superfamily, the major facilitator superfamily (MFS), the resistance nodulation division (RND) family, and the proteobacterial antimicrobial compound efflux (PACE) family (24). Earlier studies have identified that EPs are involved in functions beyond extrusion of antimicrobials in enteric pathogens such as S. flexneri, Vibrio cholerae, Listeria monocytogenes, and Salmonella typhimurium, acting as significant virulence factors (23, 24). The findings in these bacteria have shown that EPs contribute to protection against bile (25), macrophage survival (26), invasion and colonization of intestinal epithelial cells (27, 28), and aid in the expression of other virulence factors (24). AcrAB-TolC efflux pump is a well-studied EP that imparts antimicrobial resistance in *Shigella* spp., E. coli, Enterobacter spp., and Salmonella spp. isolates, part of the RND family, and is linked to the efflux of quinolones (28, 29). Other known EPs in *Shigella* spp. have also been identified for promoting fluoroquinolone resistance such as *ydhE*, *mdfA*, *marA*, and *tolC* (30–34). *Shigella* spp. and Klebsiella spp. even show the presence of *tet* efflux pumps: *tetA* and *tetB*, part of the MFS family, conferring resistance to tetracycline (34, 35).

The smallest members of the bacterial membrane transport organization are the SMR family of multidrug transporters (MDT), which are majorly concerned with the efflux of various lipophilic cationic compounds (34, 35). This family of transporters was first discovered in S. aureus about 25 years ago and are commonly located on mobile genetic components such as integrons and broad-host-range plasmids, from which they occasionally integrate into the chromosome (35, 36). There is also a presence of identifiable insertional elements near SMR genes on the chromosome or plasmid, suggesting their proficient scheme for horizontal gene transfer between different bacterial species (34, 35). SMR transporters of homodimeric and heterodimeric structures have been recognized in various Gram-negative and Gram-positive pathogens, conferring resistance to various antimicrobial agents, quaternary ammonium compounds (QAC), and lethal lipophilic cations such as DNA intercalating agents (34–40). SMR family transporters are usually 12 kDa in size, inner membrane proteins, composed generally of 100 to 140 amino acid residues, and have four transmembrane alpha-helices spanning the membrane (34–40). Extensively studied members of the family have shown that the multidrug efflux is driven by an active proton motive force, and a conserved glutamic acid residue at position 14 is critical in extruding cationic drugs (39–42). Additionally, there are three subfamilies in the SMR superfamily: small multidrug pumps (SMP), suppressors of *groEL* mutations (SUG), and the paired small multidrug resistance pumps (PSMRs) (34, 35). Numerous SMR proteins from each subfamily have been identified in bacterial pathogens, as they provide increased levels of resistance to clinically used antibiotics such as cephalosporins, β-lactams, aminoglycosides, inhibitors of dihydrofolate, and various antiseptics (34, 35). Some of the well-characterized representatives of the SMR family include the EmrE transporter in E. coli (37); SugE in *Citrobacter* and E. coli (41, 42); QacE transporter in S. aureus and Klebsiella (43–45); EbrAB and YkkCD transporters in Bacillus subtilis (46, 47); AbeS transporter in Acinetobacter *baumanni* (48); and TBsmr transporter in Mycobacterium tuberculosis (49).

SMR transporters are present in high numbers in an association with other known drug-resistance genes which display a tight genetic linkage between the two antimicrobial resistance providing systems (24, 34, 35, 50, 51). There is a rapid horizontal spread of SMR protein homologs such as that of EmrE, Qac, Gdx, and novel members of this family are being identified increasingly, making them critical transporters to be characterized in combatting the antimicrobial resistance issue in bacterial pathogens (34, 35, 50, 51). The EmrE transporter of E. coli is considered as a paradigm SMR efflux pump, having been extensively studied in the past, and is used as a foundation to study the other members of the family (37, 40, 52, 53). EmrE homologs are present in various Gram-negative bacteria, even in S. flexneri, as a 12 kDa inner membrane protein, responsible for the efflux of various antimicrobials such as erythromycin, tetracycline, methyl viologen, ethidium bromide, acriflavine, sulfadiazine, etc. (37, 39, 40). Functional studies based on the X-ray structures of EmrE, have shown that it exhibits a dual topology that is utilized for its drug efflux mechanism (53, 54). A plethora of structural, functional, and biochemical studies have been performed with EmrE over the past decades; hence, it was used as a model SMR transporter in this study to characterize the YnfA MDT of S. flexneri (37, 39, 40, 52–54).

This study explores the functional role of YnfA, an uncharacterized hypothetical protein in the S. flexneri genome. It further characterizes this efflux protein, belonging to the SMR superfamily of transporters and its involvement in contributing to *Shigella*’s resistance to various antimicrobials. To establish YnfA as an active multidrug transporter in *Shigella*, a *ynfA* knockout (KO) mutant was created and, along with the wild-type S. flexneri 1c strain (1c WT), it was subjected to further assays such as (i) MIC assay against various antimicrobial compounds using plate dilution method, (ii) determining growth and resistance patterns of the using drug sensitivity assay, and (iii) transport assays using ethidium bromide and acriflavine to examine the efflux capacities. This was followed by the *in silico* analysis of YnfA to further characterize the transporter and its homologs in different Gram-negative bacteria. Utilizing the model transporter EmrE and its already solved functional structure, the three-dimensional (3D) structure of YnfA was predicted using computational techniques. Previous knowledge from mutational studies in EmrE was also used in selecting amino acids targets in YnfA to carry out mutagenesis examination to identify the crucial amino acid residues of the protein. Similar assays as mentioned above were also carried out to also assess the effect of disrupting either one or two of the SMR efflux pumps on the resistance and transport profile of S. flexneri. The present work describes the efflux pump YnfA of the SMR family and recognizes its involvement in promoting antimicrobial resistance in S. flexneri. It ultimately provides a new drug/inhibitor target that could be exploited in the future to fight antimicrobial resistance in bacterial pathogens and aid in combatting shigellosis caused by drug-resistant strains of *Shigella*.

## RESULTS

### *In silico* analysis of YnfA and the SMR superfamily.

**FIG 1:**
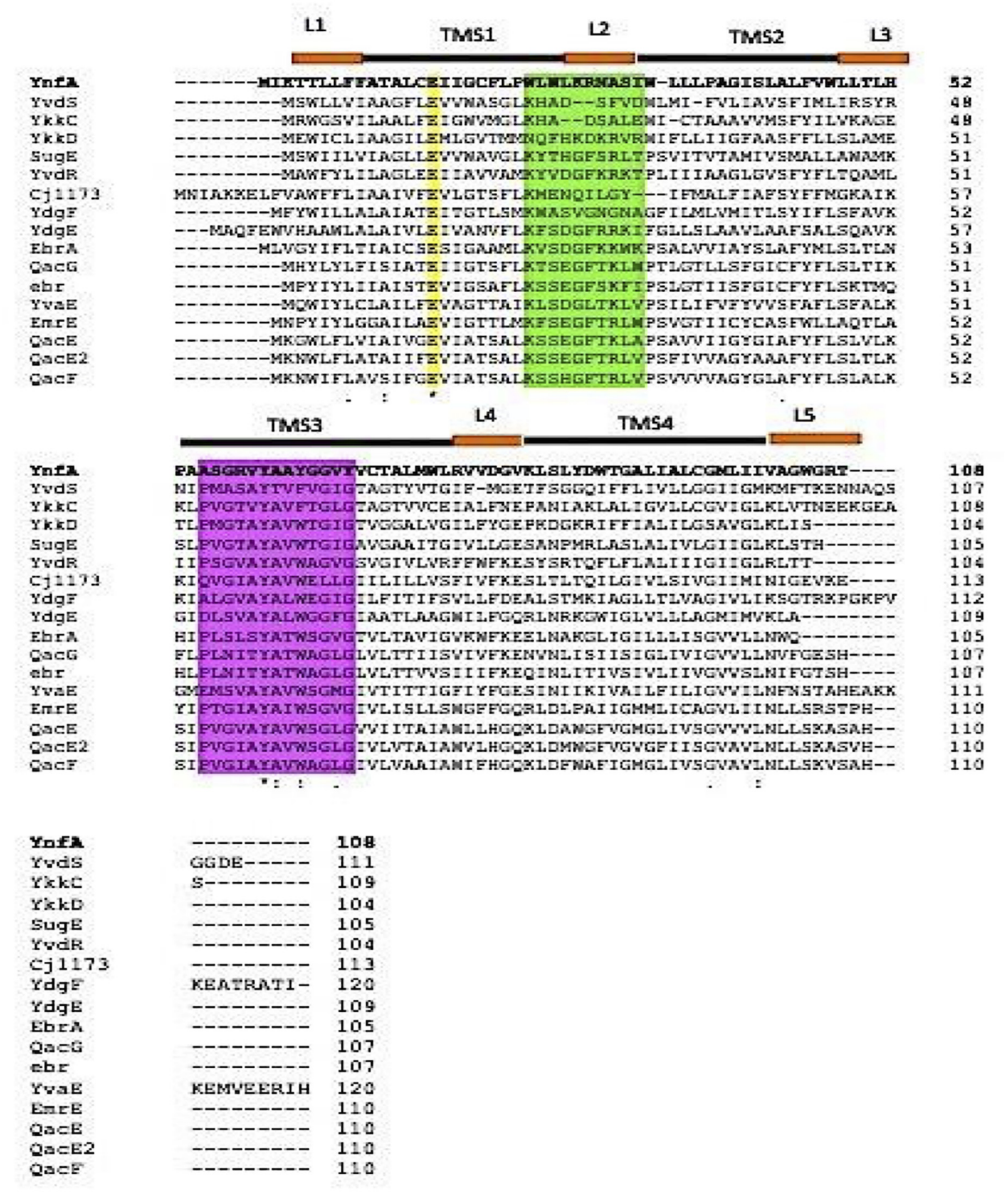
Multiple sequence alignment of the YnfA and related SMR family members. The yellow, green and pink backgrounds denote the three conserved motifs found in the SMR superfamily. Each of the four proposed YnfA transmembrane segments (TMS) are indicated by black bars, while the loops (L) are depicted as orange bars above the sequence. UniProt accession IDs of the protein members belonging to the SMR superfamily, used for the multiple sequence alignment is provided in Table S5.

**FIG 2:**
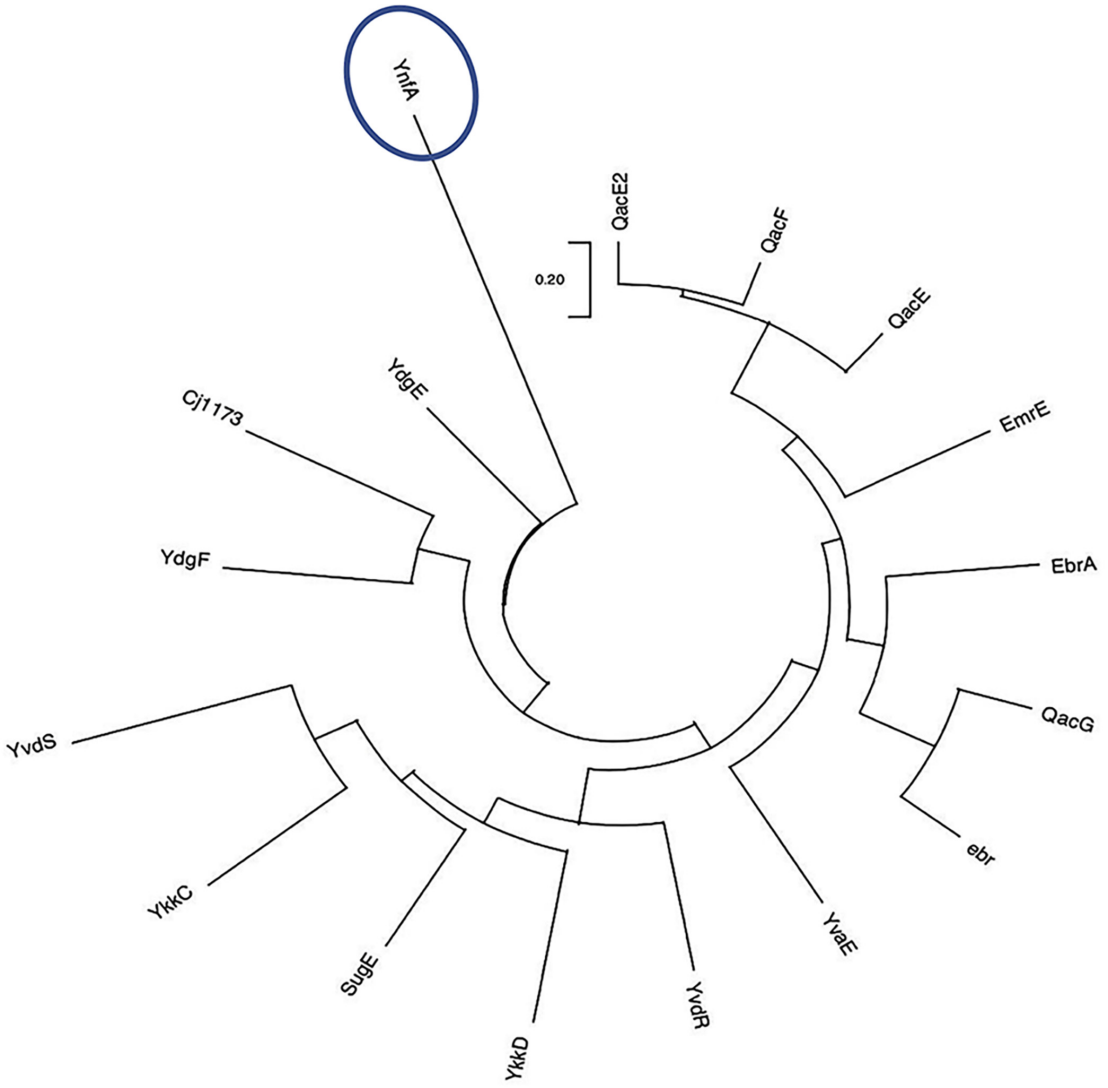
Phylogenetic tree of YnfA and the other members of the SMR superfamily. This tree is based on the multiple sequence alignment generated by Clustal-Omega (Fig. 3). The phylogenetic tree was made using the MEGA software and the Maximum Composite Likelihood method was used to calculate the evolutionary distances between the SMR members. UniProt accession IDs of the protein members belonging to the SMR superfamily, used for the multiple sequence alignment is provided in Table S5.

YnfA (ATH67966.1) was present as an uncharacterized hypothetical protein (HP) in the S. flexneri 1c genome, located at gene positions 1616397 to 1616723. YnfA was identified to be a putative multidrug transporter belonging to the SMR superfamily and is present as a 11.9 kDa integral inner membrane protein, comprised of 108 amino acids. It was predicted using the TMHMM (55) and TMpred (56) programs that YnfA consisted of four α-transmembrane helices which is a known signature of members of the SMR superfamily (Fig. S1). A multiple protein sequence alignment of YnfA sequence, with other known transporters of the SMR superfamily using Clustal-Omega (57), was performed to analyze the conserved motifs of the family members. It was observed that there is a presence of three conserved motif blocks which are believed to be indispensable for the appropriate functioning of the transporters (33–35, 52) (Fig. 1). On the basis of this multiple sequence alignment of YnfA and other SMR family proteins, a descriptive phylogenetic tree was prepared, employing the of the MEGA software (58), displaying the evolutionary distances between the YnfA and other SMR family members, and analyzed using the maximum composite likelihood method (Fig. 2). This phylogenetic analysis depicted that YnfA was a distant homolog of the other SMR family members and should be considered as a separate subfamily (YnfA family) along with the three previously known subfamilies (33–35, 52).

Additionally, it was observed that the efflux transporter YnfA is relatively widespread, and its homologs were recognized in various other Gram-negative pathogenic bacteria. The YnfA protein homologs with an E-value (<10^−4^) were aligned via Clustal-Omega to analyze the conserved amino acids. This multiple-sequence alignment was first used to check the sequence conservation of the homologs at every position by means of the WebLogo tool (59) which generates a stack of amino acid symbols. The results obtained demonstrated that the majority of amino acids are conserved in the YnfA homologs from different Gram-negative bacteria (Fig. S2). The multiple-sequence alignment was also utilized to create a phylogenetic tree for the YnfA homologs. MEGA software was used to create the tree and the maximum composite likelihood method was utilized to establish the evolutionary distances between the homologs (Fig. S3). It was observed that the YnfA protein sequence of S. flexneri closely resembles the YnfA protein of E. coli, Salmonella, *Citrobacter*, Klebsiella, and *Yersinia*, but was the most unrelated to the YnfA homolog of Staphylococcus, based on the calculated phylogenetic distances between the protein sequences.

### Structural and functional assessment of YnfA based on the EmrE transporter of E. coli.

The 3D structure of any protein is determined by various features such as its hydrogen bonding, van der Waals forces, amino acid sequence composition, and its association with the surrounding environment (60). There is an abundant presence of uncharacterized protein sequences due to the large-scale genome sequencing projects, but their structural and functional analysis is limited due to the time and cost of protein evaluation techniques such as NMR and X-ray crystallography (61). Hence, computational determination which aids in predicting protein structures can be a critical tool for functional analysis based on structure (61). Prediction of protein structures can be done by modeling and threading on a template of an already solved crystal structure of a well-studied protein (62, 63). On the basis of the homology found between the query protein sequence and the solved template, a high-resolution model of the query protein can be generated (62, 63). In this study, the I-TASSER (Iterative Threading ASSEmbly Refinement) tool (62, 63) was employed to predict the functional 3D structure of YnfA protein from S. flexneri. The I-TASSER obtained structure of YnfA was also confirmed using the AlphaFold protein structure database (64), which generated the same 3D structure for both YnfA and EmrE, validating the results.

**FIG 3:**
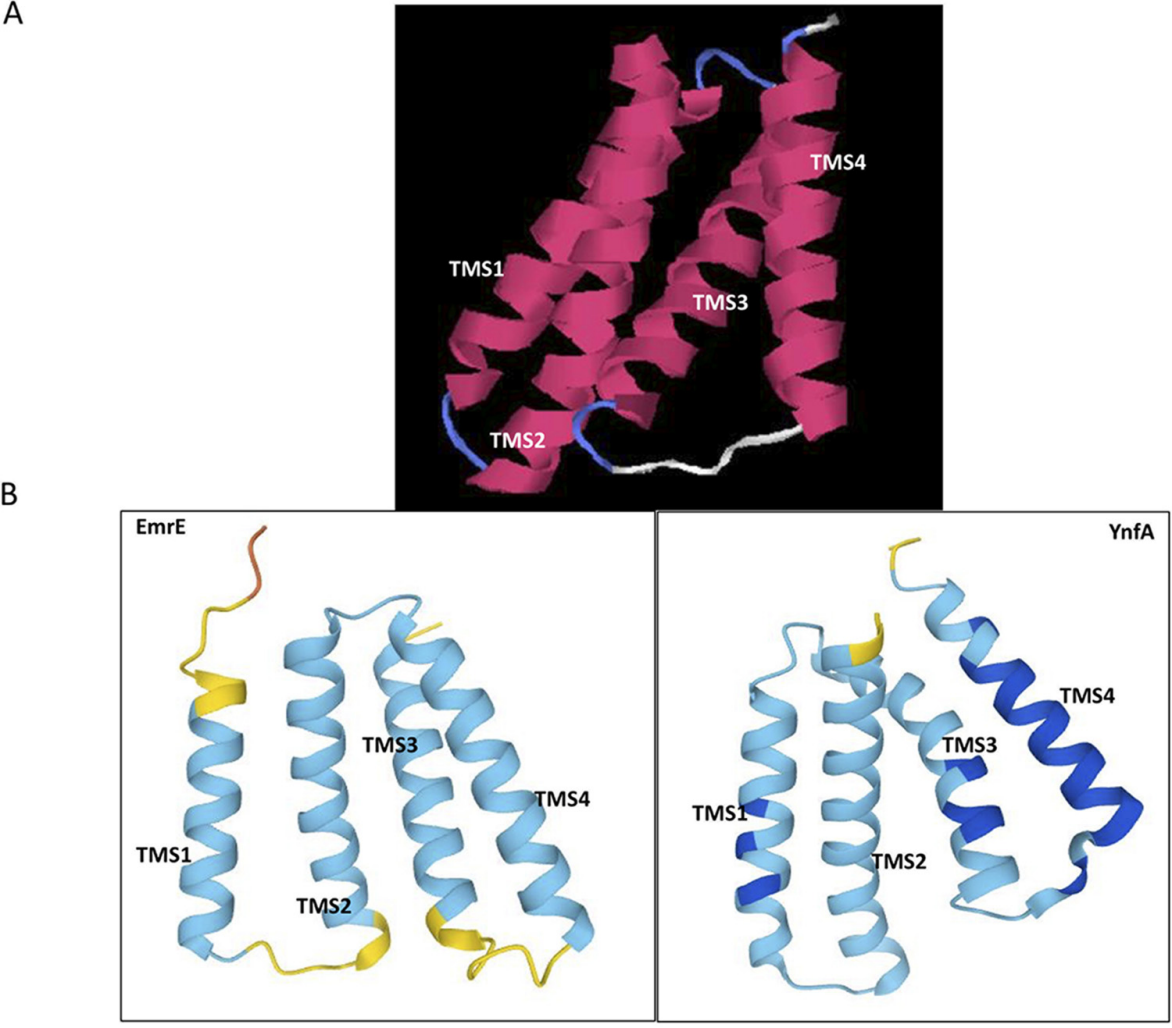
The 3D structure of YnfA protein, predicted using the I-TASSER tool and comparison to the EmrE structure using the AlphaFold protein structure database. (A) The 3D structure of YnfA computed by I-TASSER tool depicting four alpha-transmembrane helices (TMS 1 to 4). The solved structure of EmrE was used by the I-TASSER tool to thread the structure of YnfA around it as shown in the Fig. S3. (B) 3D structures of EmrE and YnfA was also generated using the AlphaFold protein structure database. The obtained structure validated the four alpha-transmembrane helical model obtained using I-TASSER tool and displayed that EmrE and YnfA transporter proteins have a similar structure.

I-TASSER used the already resolved crystal structure of EmrE transporter (PDB ID: 3b61) of E. coli as the template to predict the structure of YnfA. The predicted secondary and 3D structure of YnfA was found to be similar to the known structure of EmrE with coverage of 0.95 and a Normalized Z-score of 2.15 which is considered as good alignment and threading score (62, 63), as seen in the Fig. S4A and B. The structure is composed of four alpha-transmembrane helices, which also shows the threading alignment of YnfA and EmrE, along with the identity scores. The 3D structures of YnfA and EmrE obtained using the AlphaFold protein structure database also corroborated this predicted four alpha-transmembrane helical model as seen for the I-TASSER results (Fig. 3A and B).

SMR family members are recognized to be proton-coupled transporters and predicted to work as dimers. Extensive structural assessment of EmrE has shown that it actively works as a dimer exchanging two protons for one drug molecule per cycle (37, 39, 40, 54, 65, 66). It displays an alternating access mechanism, having at least two conformations (dual-topology) in the inner membrane which includes the inward plus outward-facing forms with both the cytoplasm or periplasm having access to the drug-binding sites (37, 39, 40, 54, 65–69). The interconversion of these forms is promoted by the drug/proton binding and aids in its efflux activity. It can be assumed here, that the YnfA transporter also follows the same dual topology model for carrying out drug efflux as EmrE. The evidence of YnfA exhibiting a dual topology model has been studied previously in E. coli by Rapp et al., 2006, using computational methods (70), but further NMR and X-ray crystallographic functional studies are needed to validate this.

### *ynfA* KO mutant shows increased susceptibility to different antimicrobials.

Evaluation of the MIC, using the microtiter plate dilution method, was carried out against various antimicrobials. Measurements of MIC against a compound for bacteria are considered to indicate the degree of susceptibility or resistance toward that specific compound (71–74). The antimicrobial agents used in this assay were selected based on the already known substrates being actively extruded by the other members of the SMR superfamily. These included antibiotics such as erythromycin, rifampicin, tetracycline, and polymyxin-B. Additionally, cationic dyes and DNA intercalating agents such as ethidium bromide, acriflavine, methyl viologen, and crystal violet were also selected. Two-fold serial dilutions of the different antimicrobial compounds were made in tryptone soy broth (TSB) media in a 96-well plate and these wells were inoculated using mid-log phase cultures (OD600 = 0.5 to 0.6). The absorbance (OD600) was measured for each well after 16 h incubation at 37°C and the MIC for each antimicrobial compound was determined as the lowermost concentration at which 90% of bacterial growth inhibition was witnessed (MIC_90_). It was seen that the *ynfA* KO mutant SFL2640 showed an increased susceptibility and lower MIC_90_ values for various antimicrobial agents used when compared to the MIC_90_ values of the 1c WT strain SFL2608 (Table 1). The *ynfA* gene was then complemented in the KO mutant strain by cloning into the pBAD_*Myc_*HisA vector to see if adding the *ynfA* gene back can restore WT phenotype. It was observed that the complemented strain SFL2643 (YnfAComp), displayed susceptibilities and MIC_90_ values similar to the 1c WT strain. These observations demonstrate that disrupting the YnfA transporter made S. flexneri more susceptible to different antimicrobials.

**TABLE 1:** MIC_90_ values of *Shigella* strains

Antimicrobial agent (range tested)^*a*^	1c WT strain (SFL2608)	SFL2640 (ΔYnfA)	SFL2643 (YnfAComp)
Erythromycin (512 to 0.5 μg/mL)	32 μg/mL	16 μg/mL	32 μg/mL
Rifampicin (512 to 0.5 μg/mL)	2 μg/mL	0.5 μg/mL	2 μg/mL
Tetracycline (512 to 0.5 μg/mL)	64 μg/mL	32 μg/mL	64 μg/mL
Polymyxin-B (512 to 0.0635 μg/mL)	4 μg/mL	0.5 μg/mL	4 μg/mL
Ethidium bromide (512 to 0.48 μg/mL)	500 μg/mL	125 μg/mL	500 μg/mL
Acriflavine (512 to 0.19 μg/mL)	31.25 μg/mL	7.8 μg/mL	31.25 μg/mL
Methyl viologen (512 to 0.19 μg/mL)	250 μg/mL	62.5 μg/mL	250 μg/mL
Crystal violet (512 to 0.19 μg/mL)	125 μg/mL	31.25 μg/mL	125 μg/mL

a 1c WT strain (SFL2608), SFL2640 (ΔYnfA), and SFL2643 (YnfAComp) were tested against 2-fold serial dilutions of various antimicrobial compounds and the MIC_90_ values were noted. Three independent repeats of the MIC assay were carried out.

### *ynfA* KO mutant displays altered growth and resistance patterns in the presence of antimicrobial compounds.

**FIG 4:**
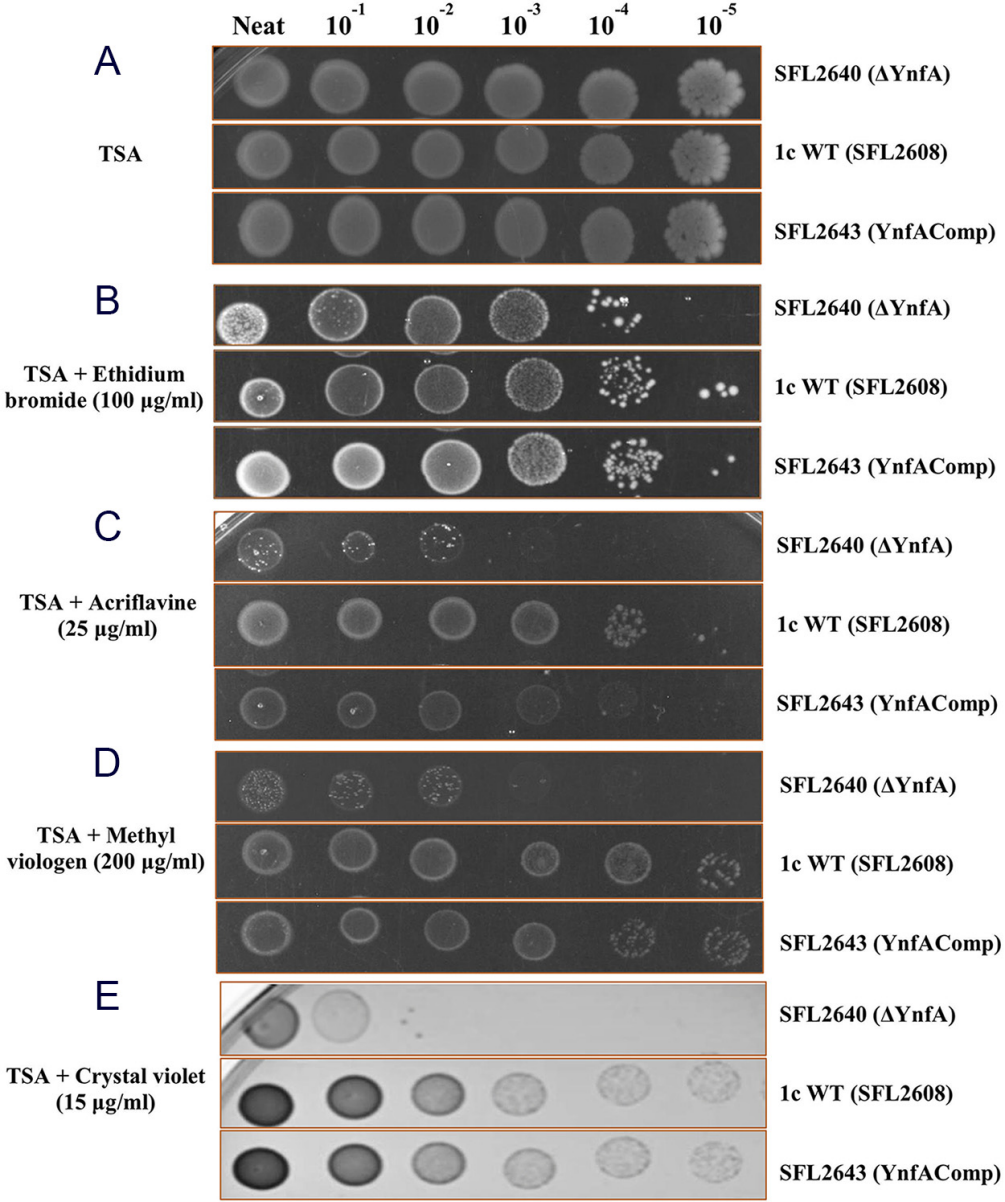
Growth phenotype of 10-fold dilutions of *Shigella* strains: 1c WT strain (SFL2608), SFL2640 (ΔYnfA), and SFL2643 (YnfAComp) on TSA plates containing different antimicrobial compounds. Cultures were grown till the mid-log phase and 10-fold serial dilutions were prepared (10^−1^ to 10^−5^). 5 μL of the neat sample, followed by each dilution was spotted on (A) TSA only, acting as the growth control, (B) 100 μg/mL EtBr, (C) 25 μg/mL Acriflavine, (D) 200 μg/mL Methyl viologen, and (E) 15 μg/mL crystal violet. Incubation of the plates was done overnight at 37°C and the experiment was performed in three independent repeats with a representative of the results shown above.

To evaluate YnfA’s role in mediating resistance in S. flexneri, the plate dilution method which has been previously used to analyze the EmrE conferred resistance in E. coli was carried out (37, 48, 52, 68). In this method, the growth of 10-fold serial dilutions of *Shigella* strains: 1c WT strain (SFL2608), SFL2640 (ΔYnfA), and SFL2643 (YnfAComp) were determined on TSA plates containing different antimicrobial compounds at a particular concentration. This assay allowed an improved way of differentiation between the KO mutant and WT strain, as it determines the growth of cells at each dilution in the presence of the antimicrobials. The compounds tested in this assay were ethidium bromide (100 μg/mL), acriflavine (25 μg/mL), methyl viologen (200 μg/mL), and crystal violet (15 μg/mL). A total of 5 μL of 10-fold dilutions of each strain was spotted on the TSB plates containing these antimicrobial compounds and growth patterns were observed after overnight incubation of the plates. A TSA plate not containing any drug was used as a control to assess the growth of all the 10-fold dilutions for each strain. A growth curve analysis of *Shigella* strains: 1c WT strain (SFL2608), SFL2640 (ΔYnfA), and SFL2643 (YnfAComp), in the absence of any antimicrobial compounds was also carried out. This was done to assess if there was any fundamental growth defect caused by deleting the *ynfA* gene and confirming if the growth variations seen in the presence of antimicrobials was purely due to disrupting the YnfA efflux pump. It was observed that SFL2640 (ΔYnfA) KO mutant showed increased sensitivity and an altered growth pattern on the various compounds tested. In comparison, the 1c WT strain (SFL2608) and the complemented strain (SFL2643/YnfAComp) showed lesser sensitivity and grew better on TSB plates containing these compounds as can be seen in Fig. 4. There was no significant difference observed in the growth patterns of the three *Shigella* strains in the absence of any antimicrobial compounds as seen in the growth curve analysis ruling out any growth defect in the absence of antimicrobials (Fig. S5) and the control 10-fold dilutions spots on a TSA plate (Fig. 4A). These observations indicated that, after deleting the *ynfA* gene, disrupting the efflux pump makes *Shigella* more sensitive to the antimicrobial drugs and affects its overall growth on these compounds.

### *ynfA* KO mutant demonstrates reduced transport activity.

To analyze the transport capabilities of the WT, complement, and KO mutant S. flexneri strains: 1c WT strain (SFL2608), SFL2640 (ΔYnfA), and SFL2643 (YnfAComp), a fluorescence-based transport assay was carried out using ethidium bromide (EtBr) and acriflavine. These assays using fluorescent dyes such as EtBr, acriflavine, Nile red, or other fluorescently labeled compounds are well established and have been used in various bacteria to monitor efflux activities (75–78). The main principle of this fluorometric assay is that when fluorescent compounds are present at sublethal doses, they enter the bacterial cell by passive diffusion, but can only be eliminated by active efflux systems (75–78). Hence, estimating the intracellular concentration of the fluorescent dye can be directly associated with the activity of efflux machinery (75–78). EtBr, a DNA intercalating agent, is known to emit weak fluorescence in solution (outside the bacterial cell) and the fluorescence intensity increases when the concentration of EtBr increases inside the bacterial cells. Thus, bacterial cells with a lower efflux capability will have a greater fluorescence as more intracellular levels of EtBr would be present (75, 76). Acriflavine, which is a commonly used topical antiseptic, has also been previously used as a fluorescent dye to investigate efflux activities (77, 79–81). Unlike EtBr, the fluorescence intensity of acriflavine is low, when inside the bacterial cells and increases when in the solution (outside the cells) (77, 79–81). It has been shown in earlier studies that acriflavine binds to DNA when inside the cell resulting in an quenching of its fluorescence intensity (77, 79–81). Hence, cells with a lower efflux activity will have diminished fluorescence, as more intracellular levels of acriflavine will be present and the efflux of acriflavine from cells, causing its dissociation from DNA will increase the fluorescence (77, 79–81).

**FIG 5:**
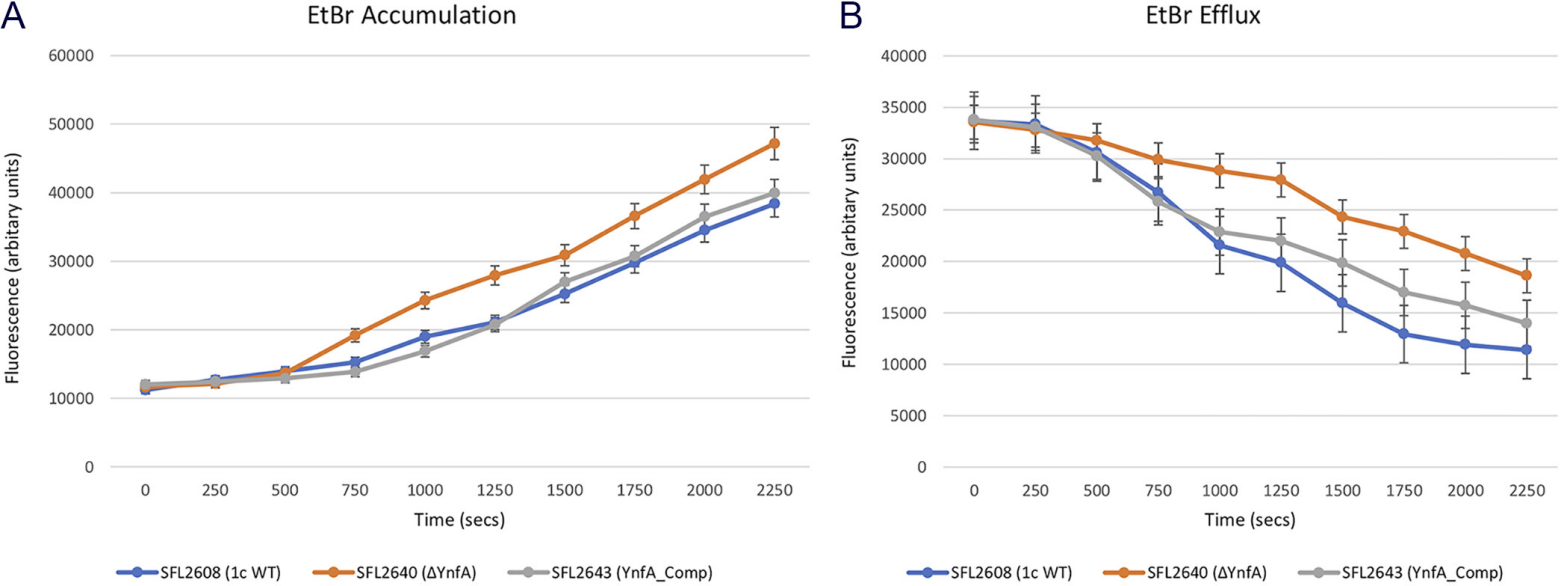
Ethidium bromide transport by *Shigella* strains. A fluorometric assay was carried out with 1c WT strain (SFL2608), SFL2640 (ΔYnfA), and SFL2643 (YnfAComp), in which cells were grown until mid-log phase, collected in PBS buffer and loaded with 2 μg/mL of EtBr. (A) Accumulation and (B) efflux were monitored after adding 0.4% vol/vol of glucose to energize the cells at time zero. Fluorescence was measured at 37°C with an excitation wavelength of 518 nm and an emission wavelength of 605 nm. The results are based on three autonomous experiments and the error bars represent the standard deviation.

**FIG 6:**
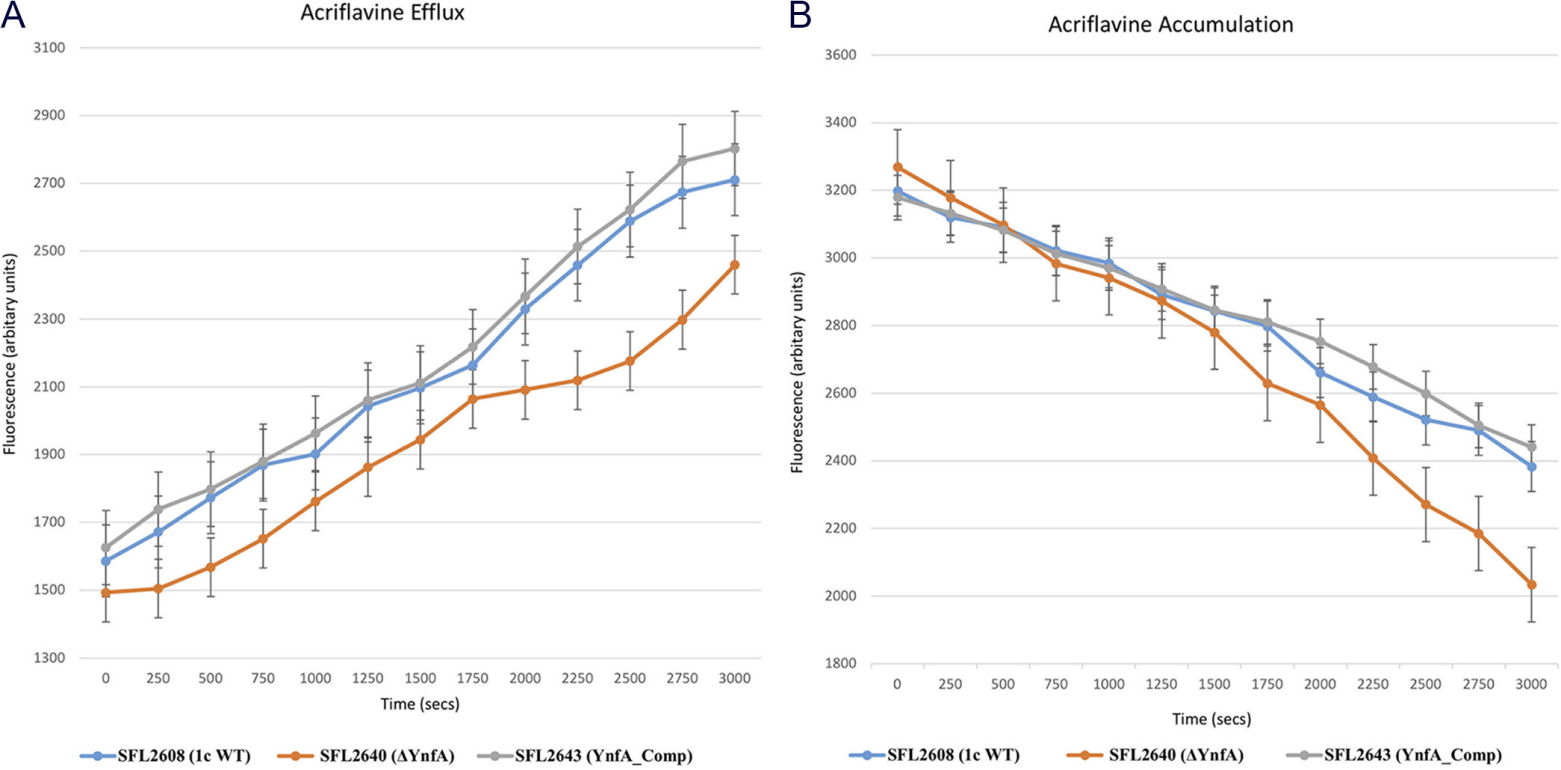
Acriflavine transport by *Shigella* strains. A fluorometric assay was carried out with 1c WT strain (SFL2608), SFL2640 (ΔYnfA), and SFL2643 (YnfAComp), in which cells were grown until mid-log phase, collected in PBS buffer and loaded with 5 μM acriflavine. (A) Accumulation and (B) efflux were monitored after adding 0.4% vol/vol of glucose to energize the cells at time zero. Fluorescence was measured at 37°C with an excitation wavelength of 450 nm and an emission wavelength of 510 nm. The results are based on three autonomous experiments and the error bars represent the standard deviation.

Time course fluorescence-based assays for both accumulation and efflux were carried out using EtBr and acriflavine with the *Shigella* strains. It was observed that the accumulation of both EtBr and acriflavine was much higher in the KO mutant SFL2640 (ΔYnfA) when compared with the 1c WT (SFL2608) and *ynfA* complement strain SFL2643 (YnfAComp) (Fig. 5A and 6A). Subsequently, in the efflux assays with EtBr and acriflavine, a similar trend was noted, wherein the KO mutant SFL2640 (ΔYnfA) displayed a reduced efflux activity in comparison with the activity seen in the 1c WT (SFL2608) and *ynfA* complement strain SFL2643 (YnfAComp) (Fig. 5B and 6B). The lower efflux activity and, hence, a higher accumulation of EtBr and acriflavine in the *ynfA* KO mutant is possibly due to the disruption of the YnfA transporter, which leads to an overall reduction of the efflux activity of the bacteria. Transport assay results sit consistent with the MIC_90_ and drug sensitivity observations seen for the *ynfA* KO mutant signifying that the YnfA transporter like the other members of the transporter family is indeed involved in extruding antimicrobial compounds and conferring resistance in *Shigella.*

### Mutagenesis study of the YnfA transporter.

The protein sequences of YnfA and EmrE showed a 29.82% similarity with an E-value of 0.53, when an NCBI-protein BLAST was carried out with modified parameters (higher threshold level: 0.6). Following this, amino acid analysis of the YnfA protein using Clustal-Omega multiple sequence alignment with the E. coli EmrE protein was done and this alignment showed the conserved, weakly similar, and dissimilar amino acids among both the proteins. It was seen that most of the essential amino acid residues were conserved, as was the case in most transporters of the SMR family (Fig. S6). Mutational studies done with EmrE and other well-studied members of the SMR family have identified amino acid residues that are crucial for proper functioning (39, 69, 82). One is Glutamic acid residue at the 14th position (Glu-14) which has been recognized as the major player in EmrE’s transport and binding site for the substrates and protons (39, 83–85). There is also a presence of a set of five amino acids near the Glu-14 residue (Leu-7, Ala-10, Ile-11, Gly-17, and Thr-18) in EmrE, that are known to be important for the proper functioning of the transporter, being involved in the recognition and translocation of substrate and protons (86). Recognizing these important amino acids in EmrE (highlighted in yellow in Fig. S6), helped in shortlisting the targets for the subsequent site-directed mutagenesis in YnfA. Amino acid targets were selected based on prior knowledge about the essential and critical amino acids of EmrE and other studied SMR family transporters. Some of the amino acid targets were also chosen based on them being highly conserved in YnfA homologs from other Gram-negative bacteria, which was worked upon using a consensus YnfA sequence generated using the Weblogo tool (Fig. S2). Founded on this, nine mutagenesis targets were selected as highlighted in green in Fig. S6 and detailed in Table S1.

YnfA mutants were cloned into the pBAD_*Myc*_HisA plasmid and Western blot with anti-HisA antibody was used to confirm for appropriate protein expression. All YnfA mutants were expressed appropriately after ensuring equal total protein loading when compared with the WT YnfA protein SFL2643 (YnfAComp) as seen in Fig. S7A and B. YnfA is a 11.9 kDa protein and the protein bands were observed below the 15 kDa band of the prestained protein ladder. Membrane proteins typically with transmembrane helices display slight gel shifts and migrate anomalously on SDS-PAGE gels (87, 88). They are known to run either higher or lower relative to their formula molecular mass (87, 88). Consequently, it is seen that YnfA runs slightly higher in the Western blot, close to the 15 kDa band of the protein ladder. Furthermore, to analyze the consequence of amino acid mutations on the functioning of the YnfA transporter, MIC_90_ determination and transport assays using ethidium bromide and acriflavine as the substrates were carried out. Ethidium bromide and acriflavine were selected as substrates for the functional assays as these are well-known compounds being transported by members of the SMR family, as is the case for YnfA. Empty pBAD_*Myc*_HisA (SFL2662) vector was considered as the negative control for Western blotting and the other experimental assays performed.

### Resistance profiling of YnfA mutants using microtiter plate assays.

**FIG 7:**
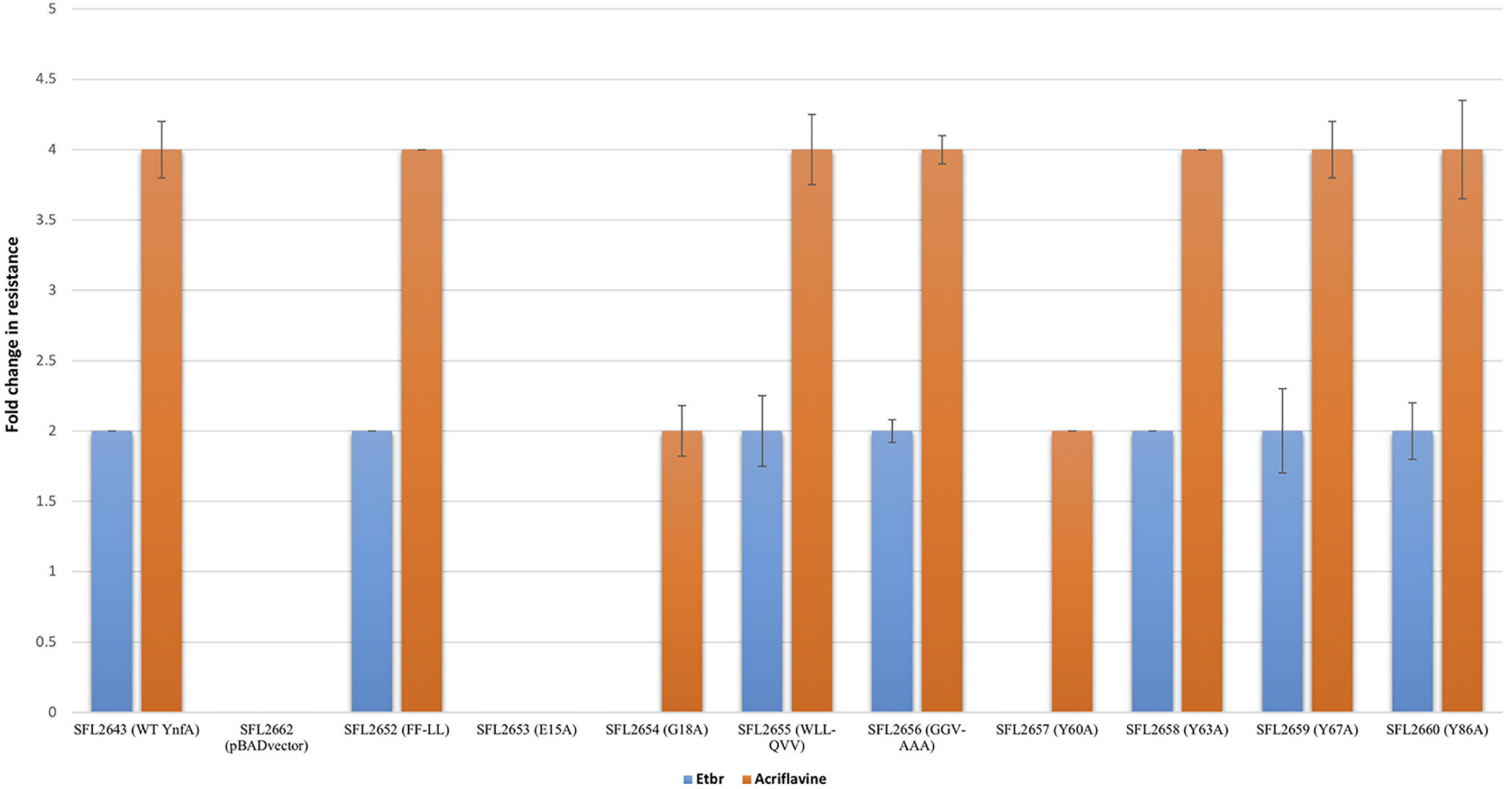
Fold change in resistance for *Shigella* strains expressing WT YnfA and site-directed YnfA mutants against EtBr and acriflavine. The relative fold change of MIC_90_ of *Shigella* strains expressing WT and mutant YnfA proteins is presented compared to the MIC of *Shigella* strain carrying the empty pBAD vector (SFL2662). Cultures were grown till the mid-log phase and added to 96-well microtiter plates containing 2-fold serial dilutions of ethidium bromide and acriflavine. Results were obtained following overnight growth at 37°C and are based on independent experiments carried out in triplicates with the error bars representing the standard deviation of the data.

The assessment of changes in resistance of the *Shigella* strains harboring the nine site-directed mutants of YnfA: SFL2652 (FF-LL), SFL2653 (E15A), SFL2654 (G18A), SFL2655 (WLL-QVV), SFL2656 (GGV-AAA), SFL2657 (Y60A), SFL2658 (Y63A), SFL2659 (Y67A), and SFL2660 (Y86A), along with the WT YnfA protein (SFL2643) and the pBAD_*Myc*_HisA empty vector (SFL2662) which was used as the negative control. EtBr and acriflavine, which are two common SMR substrates, were chosen to determine the resistance levels of these mutants against them using a microtiter MIC assay. The *Shigella* strains were subjected to serial 2-fold dilutions of EtBr and acriflavine, prepared in a 96-well plate, and absorbance (OD600) was measured of each well after 16 h incubation at 37°C. The MIC for each antimicrobial compound was determined as the lowermost concentration at which 90% of bacterial growth inhibition was detected (MIC_90_). The results shown in Fig. 7 are presented as a fold increase in resistance relative to the results obtained for the strain carrying the empty pBAD vector (SFL2662). It was seen that YnfA mutants SFL2653 (E15A), SFL2654 (G18A), and SFL2657 (Y60A) displayed a decreased resistance to EtBr and acriflavine when compared with the WT YnfA protein (SFL2643), which on the other hand, showed a 2-fold greater resistance to EtBr and 4-fold greater resistance to acriflavine. The other mutants,—SFL2652 (FF-LL), SFL2655 (WLL-QVV), SFL2656 (GGV-AAA), SFL2658 (Y63A), SFL2659 (Y67A), and SFL2660 (Y86A)—showed no changes in their resistance profile when compared with the WT YnfA protein (SFL2643).

### Determining transport activity of the YnfA mutants.

**FIG 8:**
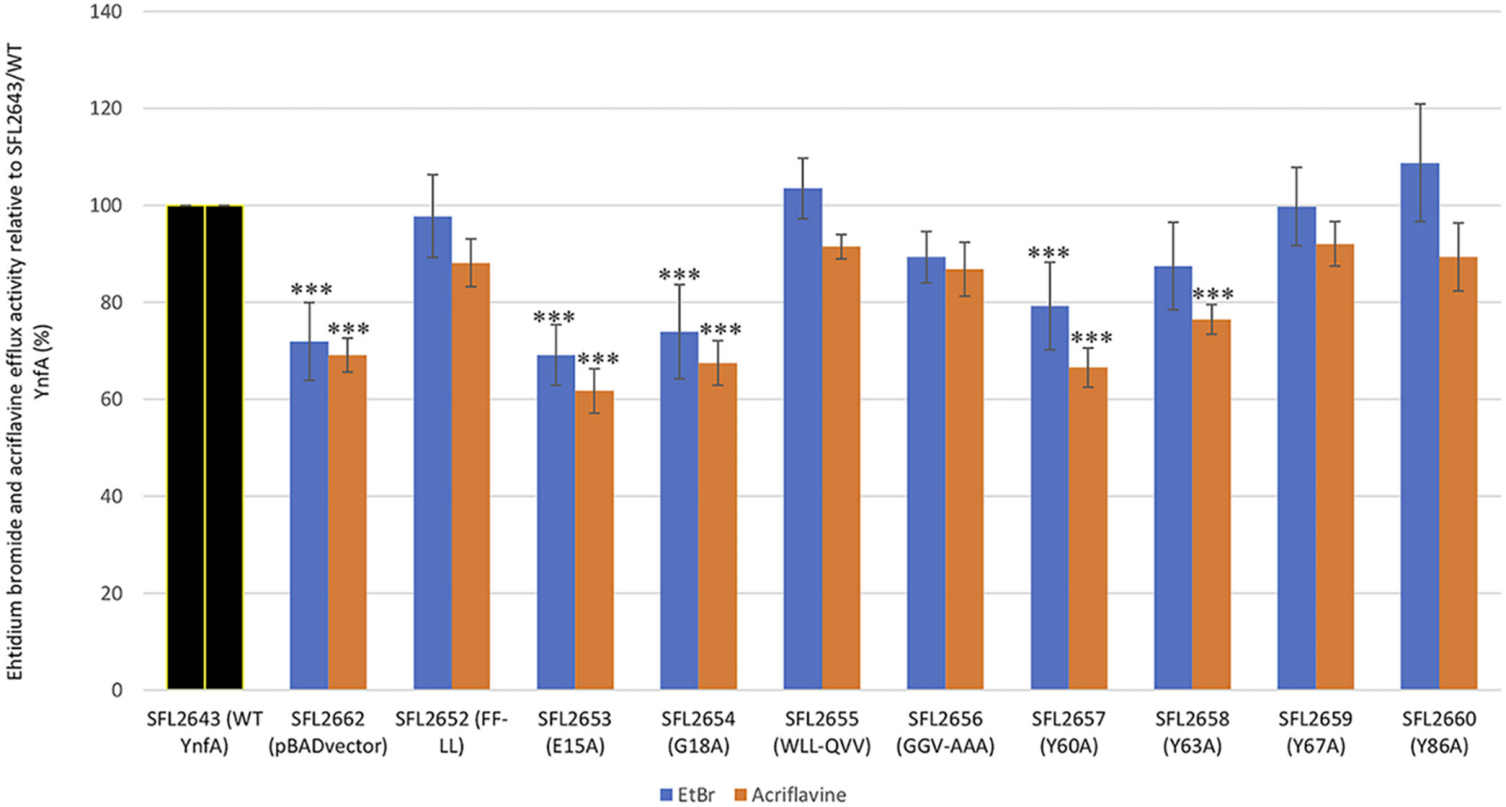
Efflux transport of acriflavine and EtBr in *Shigella* strains expressing the WT and mutant YnfA proteins. Percentage of efflux activity of acriflavine and EtBr for the YnfA mutants was calculated compared to the WT YnfA protein (SFL2643), which indicates 100% export and is represented as the black bar. The *Shigella* strain carrying the empty pBAD vector (SFL2662) is included as a negative control. Error bars represent the standard deviations of three biological replicates. The asterisks represent that the variance seen were statistically significant (*P* < 0.05). The percentage of efflux activity of *Shigella* strains in acriflavine (5 μM) is shown in orange and the percentage of efflux activity of *Shigella* strains in EtBr (2 μg/mL) is shown in blue.

Evaluation of the transport activity of the YnfA mutants was carried out using a fluorescence-based transport assay with EtBr and acriflavine as the substrates. The efflux capabilities of the nine YnfA mutants, SFL2652 (FF-LL), SFL2653 (E15A), SFL2654 (G18A), SFL2655 (WLL-QVV), SFL2656 (GGV-AAA), SFL2657 (Y60A), SFL2658 (Y63A), SFL2659 (Y67A), and SFL2660 (Y86A), along with the WT YnfA (SFL2643) and the empty pBAD_*Myc*_HisA vector (SFL2662), which was considered as the negative control, was determined. Fluorescent compounds like EtBr and acriflavine enter the bacterial cell through passive diffusion, however, can only be extruded by efflux systems of the bacteria. Evaluating the intracellular concentration of the fluorescent compound can directly give an estimate of the efflux activity in the bacteria. The results shown in Fig. 8 are represented as the percentage of efflux activity in acriflavine and EtBr seen relative to the WT YnfA protein (SFL2643), which is indicative of 100% export.

In the case of acriflavine efflux assay, it was observed that four YnfA mutants showed an impaired efflux activity when compared with the WT YnfA protein and was similar to the transport activity seen in the negative control empty pBAD vector strain (SFL2662). These were SFL2653 (E15A) with 30% reduction in EtBr efflux and 38% reduction in acriflavine efflux activity; SFL2654 (G18A) with 25% reduction in EtBr efflux and 30% reduction in acriflavine efflux activity; SFL2657 (Y60A) with 22% reduction in EtBr efflux and 35% reduction in acriflavine efflux activity; and finally SFL2658 (Y63A) with no reduction in EtBr efflux and 22% reduction in acriflavine efflux activity The other five mutants, SFL2652 (FF-LL), SFL2655 (WLL-QVV), SFL2656 (GGV-AAA), SFL2659 (Y67A), and SFL2660 (Y86A), showed comparable efflux activity to the WT YnfA protein (SFL2643). A parallel transport assay carried out with EtBr as the substrate showed similar results, in which YnfA mutants SFL2653 (E15A), SFL2654 (G18A), and SFL2657 (Y60A) displayed a significantly reduced efflux capacity, and the mutant SFL2658 (Y63A) demonstrated a minor decrease in efflux activity when paralleled to the WT YnfA protein. The other five mutants, SFL2652 (FF-LL), SFL2655 (WLL-QVV), SFL2656 (GGV-AAA), SFL2659 (Y67A), and SFL2660 (Y86A), showed no change in their efflux activity compared with the WT YnfA protein (SFL2643). Hence, it can be concluded, with the results obtained from the above three assays, that amino acid residues such as E-15, G-18, Y-60, and Y-63 play crucial roles in the YnfA protein in terms of its transport activity and resistance conferment. The other amino acids tested here such as the FF pair, triplets WLL and GGV, along with the tyrosine residues Y-67 and Y-86, showed minor to no effect on YnfA’s transport activity and ability to promote resistance in *Shigella* strains. However, additional structural assessment of YnfA can validate these observations and also expand our knowledge of all the critical amino acid residues in YnfA with their respective functions (84–86, 89–91).

### Analyzing the effect on the overall bacterial efflux activity caused by the disruption of EmrE and YnfA transporters in *Shigella*.

Even though the EmrE and YnfA transporters do not show a significant overall similarity in their protein sequences, there is the occurrence of extremely conserved amino acid residues in both proteins. Both YnfA and EmrE belong to the SMR superfamily, are hydrophobic, and are considered as H+/drug antiport systems which are expected to harness the proton motive force (PMF) through the bacterial membrane by swapping two protons against one substrate molecule (33–35). The 3D-protein structure of YnfA closely resembles that of the EmrE and is believed to be present as a homodimer in the inner membrane, using a similar dual topology transport mechanism like the EmrE (37, 38, 40, 54, 66–69). The multidrug transporter EmrE is present as a 12 kDa protein in E. coli and confers resistance to ethidium bromide, acriflavine, tetraphenylphosphonium (TPP+), methyl viologen, and various other compounds with high affinities (37, 82). It was observed in the experimental analysis carried out in this study that YnfA seems to be involved in extruding similar compounds such as ethidium bromide and acriflavine, just like the EmrE transporter. Thus, it can be assumed that YnfA is a distant homolog of EmrE, and both the transporters contribute to the efflux activity and antimicrobial resistance seen in S. flexneri. However, more research needs to be carried out in the structural and functional assessment of YnfA in Gram-negative bacteria to evaluate its role as a multidrug transporter promoting antimicrobial resistance.

The EmrE (ATH68298.1) transporter is also present in S. flexneri 1c serotype, with 100% sequence similarity with the E. coli EmrE protein. The *emrE* gene (2014103 to 2014435) in *Shigella* is located far downstream of the *ynfA* gene (1616397 to 1616723); hence, both are present as individual genes and are not cotranscribed. Consequently, it was decided to delete the *emrE* and *ynfA* genes from S. flexneri serotype 1c (SFL2608) to analyze the effects of creating single and double KO mutants: SFL2640 (ΔYnfA), SFL2644 (ΔYnfA + ΔEmrE), and SFL2661(ΔEmrE). These KO mutants were subjected to fluorescence-based transport assays with acriflavine and EtBr, to evaluate the changes in transport activity due to the disruption of either EmrE or YnfA protein and additionally disrupting both the transporter proteins.

**FIG 9:**
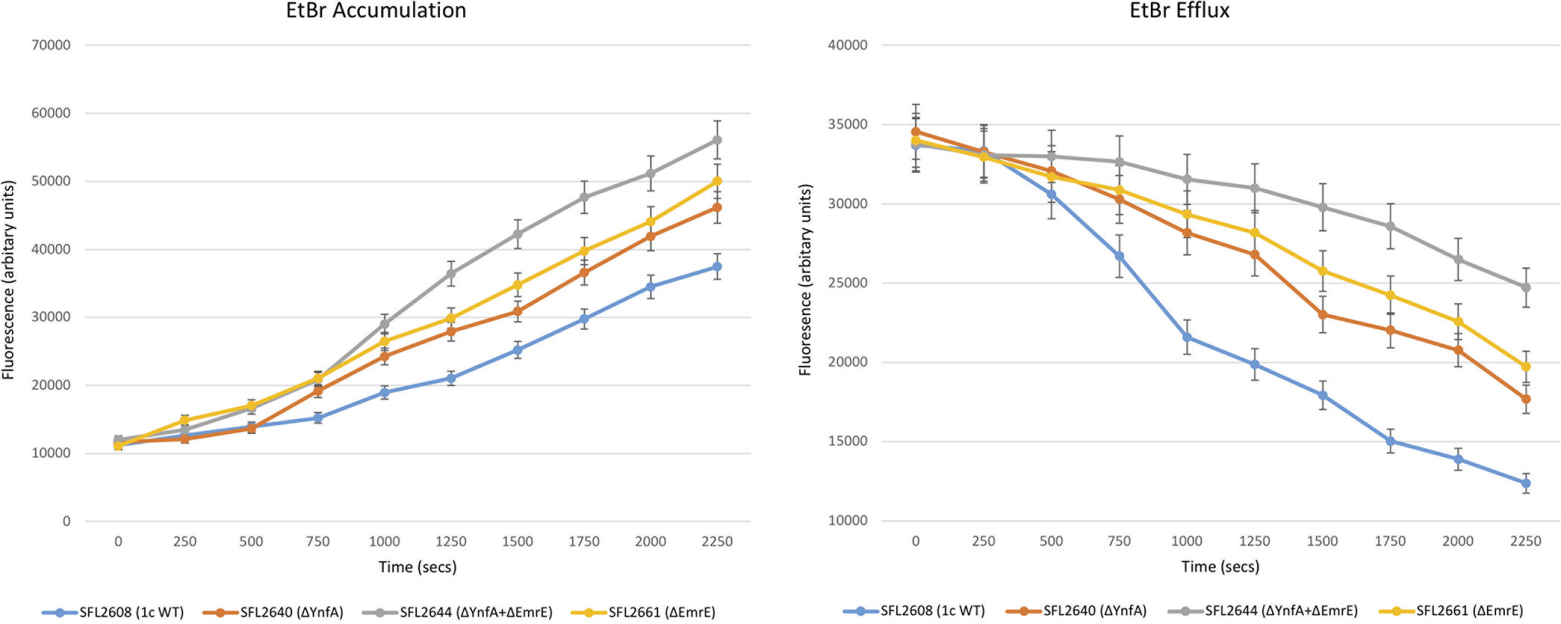
Ethidium bromide transport by *Shigella* strains. A fluorometric assay was carried out with 1c WT strain (SFL2608) and KO mutants: SFL2640 (ΔYnfA), SFL2644 (ΔYnfA + ΔEmrE), and SFL2661(ΔEmrE). Cells were grown until the mid-log phase, collected in PBS buffer and loaded with 2 μg/mL of EtBr. Accumulation and efflux were monitored after adding 0.4% vol/vol of glucose to energize the cells at time zero. Fluorescence was measured at 37°C with an excitation wavelength of 518 nm and an emission wavelength of 605 nm. The results are based on three autonomous experiments and the error bars represent the standard deviation.

**FIG 10:**
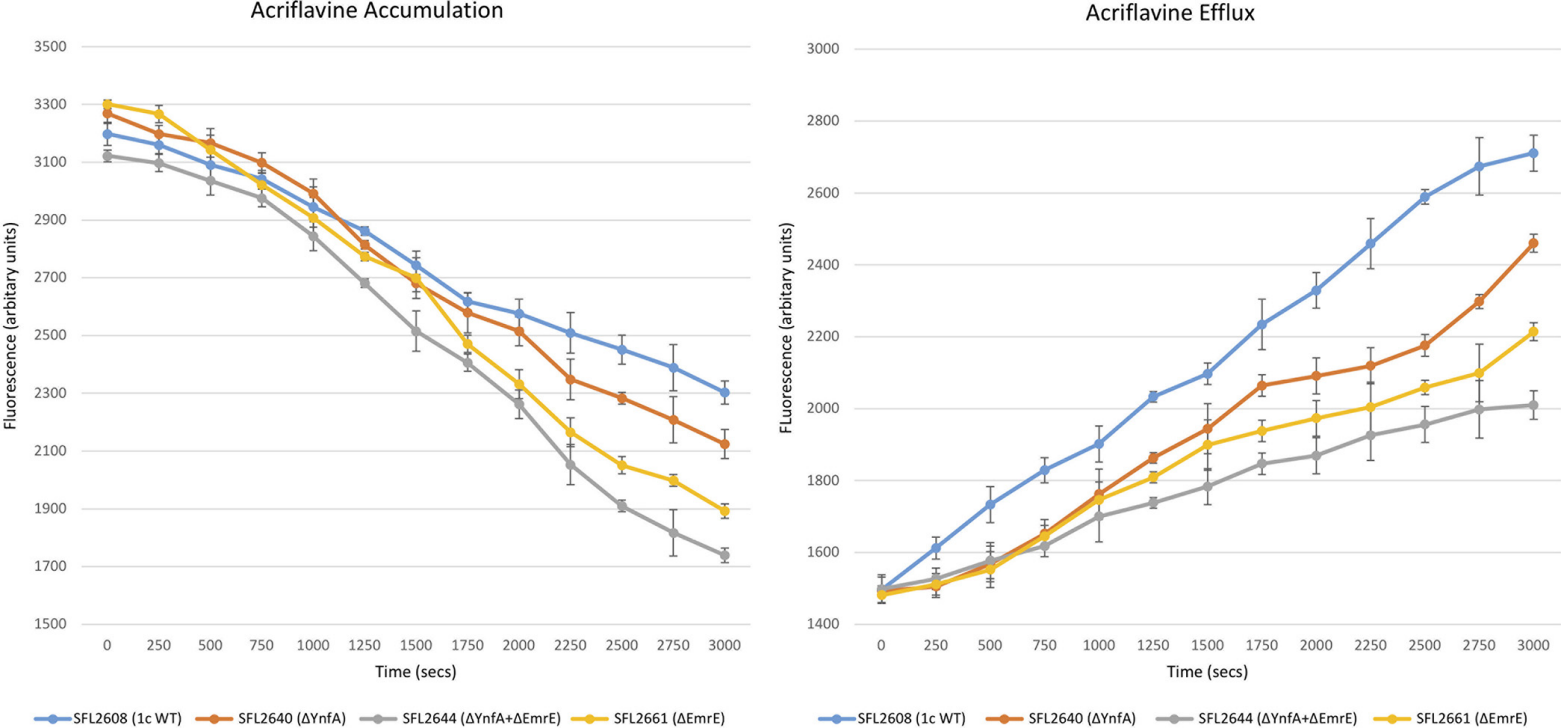
Acriflavine transport by *Shigella* strains. A fluorometric assay was carried out with 1c WT strain (SFL2608) and KO mutants: SFL2640 (ΔYnfA), SFL2644 (ΔYnfA + ΔEmrE), and SFL2661(ΔEmrE). Cells were grown until the mid-log phase, collected in PBS buffer and loaded with 5 μM acriflavine. Accumulation and efflux were monitored after adding 0.4% vol/vol of glucose to energize the cells at time zero. Fluorescence was measured at 37°C with an excitation wavelength of 450 nm and an emission wavelength of 510 nm. The results are based on three autonomous experiments and the error bars represent the standard deviation.

It was observed that the double KO mutant SFL2644 (ΔYnfA + ΔEmrE) had a significantly reduced efflux activity of acriflavine and EtBr when compared with the 1c WT (SFL2608) and the single KO mutants SFL2640 (ΔYnfA) and SFL2661(ΔEmrE) (Fig. 9 and 10). It was also seen that deleting *emrE* had more effect on the bacterial transport activity compared with deleting the *ynfA* gene. The SFL2661(ΔEmrE) KO mutant showed lesser transport activity of acriflavine and EtBr compared with the KO mutant SFL2640 (ΔYnfA) as seen in Fig. 9 and 10. These results indicate that EmrE and YnfA are both responsible for the overall bacterial efflux action by extruding similar substrates, although EmrE appears to be better at transporting acriflavine and EtBr in comparison with YnfA. This apparent superior transport activity seen in EmrE when compared with YnfA could also be due to the possible differential expression of both the genes in the *Shigella* genome. The presence of two transporters exhibiting transport of similar substrates can be explained by genetic redundancy which is commonly seen in bacteria, where two or more genes contribute to the same phenotype (92). This kind of functional redundancy wherein two proteins perform parallel biochemical functions and have comparable substrate affinities confers an added advantage to bacterial pathogens where either protein can compensate in the absence of the other (92). As SMR proteins are mostly found on mobile genetic components like integrons and plasmids and/or have familiar insertional elements near their genes, it is also possible that both the genes *emrE* and *ynfA* integrated into the bacterial chromosome via a plasmid/integron thereby conferring antimicrobial resistance in S. flexneri (33–37).

## DISCUSSION

Shigellosis caused by *Shigella* spp. remains a common cause of diarrheal deaths especially in children below 5 years of age and in countries with poor socio-economic conditions (1–4, 93). Although *Shigella* genus consists of four species, the majority of the cases worldwide are initiated by S. flexneri and S. sonnei (1). To prevent the spread of *Shigella* and the high number of shigellosis cases each year, a globally effective vaccine is needed (5–7). Extensive research work is under way to develop a *Shigella* vaccine but the current absence of one makes us heavily dependent on the available treatment options (6). Antimicrobial therapy along with oral rehydration is the recommended treatment by the WHO for shigellosis cases (8, 10–12); however, the overuse or incorrect use of antibiotics can increase the issue of antimicrobial resistance in *Shigella* (14–16, 94–96). Infections with resistant *Shigella* strains increase the time taken and overall cost of medical care needed which is even more problematic in developing and underdeveloped countries (14–16, 94–96). Resistant strains of S. flexneri and S. sonnei have already been reported over the past decades, which have shown resistance to antibiotics like ciprofloxacin, azithromycin, ceftriaxone, chloramphenicol, tetracyclines, etc., which are part of the first and second line of treatment recommended by WHO (14–16, 94–96). The generation of antimicrobial resistance in bacteria can be due to various intrinsic and acquired resistance mechanisms (18, 19), out of which the extrusion of drugs by active efflux pumps is one of the primary factors (18, 19). Bacterial cells use powerful pumping machines composed of one or more protein components traversing the cell membrane to remove toxic molecules (18, 19). Multidrug efflux pumps are being extensively studied in their role to confer resistance due to their widespread distribution in numerous bacterial species and wide range of substrate specificity (18, 19, 25).

This present study characterizes the YnfA transporter, which was present as an 11.9 kDa hypothetical protein (HP) in the S. flexneri 1c genome with no known functions. The initial *in silico* analysis carried out and identified this HP as an inner membrane protein belonging to the SMR superfamily, and the putative function predicted was drug transport. Following this, the *ynfA* gene was deleted from *Shigella* genome and subjected to various phenotypic assays to experimentally validate its functional role. Functional assays carried out in this study aided in validating YnfA as an active SMR drug-transporter and identified its role in conferring antimicrobial resistance in S. flexneri. There are five major families of bacterial multidrug transporters, out of which the SMR protein family consists of small inner-membrane proteins that are usually 100 to 140 amino acids long and have four transmembrane segments (25). The SMR family, discovered 25 years ago, continues to grow rapidly as more and more homologs are being identified and functionally characterized (33–35). The well-studied members of this family such as EmrE, SugE, and YdgE/F, and QacC are used as the foundation to study newer identified members and evaluate their transport activity (33–35, 52). Numerous structural and functional assessment of EmrE transporter in E. coli has been carried out, which was first identified in 1992 (37–40, 53, 82). Due to this, EmrE is considered an archetype for the SMR protein family and has been utilized as a model to study the evolution of membrane proteins (53). In this study as well, EmrE was used as a paradigm for SMR transporters, and all functional analyses carried out with the YnfA protein were based on it.

Bioinformatic analysis of the YnfA protein sequence predicts the presence of four alpha-helical transmembrane segments, identifying it as a member of the SMR superfamily (33–35). Multiple-sequence alignment of YnfA with other known members of the SMR family and the resulting phylogenetic tree suggested that it should be assigned as a separate subfamily along with three already known subfamilies (PSMR, SMP, and SUG) (33–35). The multiple sequence alignment also helped in identifying conserved motifs in various members of the SMR family. Following this, YnfA homologs (E > 10^−4^) were identified in numerous Gram-negative bacterial pathogens. A multiple-sequence alignment of these homolog proteins was done and a phylogenetic tree showing the evolutionary distance in between the homologs was created. The multiple-sequence alignment also resulted in a consensus protein sequence showing the most conserved amino acids in the YnfA homologs. As there is a lack of solved protein structures of the efflux transporters belonging to the SMR family, the majority of the structural analysis is carried out using computational prediction methods. The I-TASSER tool and AlphaFold protein structure database was used for computational analysis of the YnfA protein structure. I-TASSER utilized the solved EmrE protein structure as a template to thread the YnfA protein sequence around it and the resulting structure consisted of four alpha-transmembrane helices, which was further validated using AlphaFold. A multiple-sequence alignment of YnfA and EmrE protein was done employing Clustal-Omega and the amino acids conserved in both the proteins were identified. Even though YnfA and EmrE seem to be distant homologs of each other, it can be assumed that YnfA’s transport machinery is comparable with the EmrE transporter due to their similarity in structures and conserved amino acid residues. YnfA, seemingly just like EmrE, is also present as a homodimer and displays a dual topology to direct the transport and due to this arrangement, the conserved Glu15 amino acid residue sits in the middle of both the protein topologies and forms the binding domain for the substrate and protons (39, 83–85).

Functional assessment of the YnfA protein was carried out using the *ynfA* KO mutant (SFL2640), along with the S. flexneri 1c WT strain (SFL2608) and a *ynfA* complement strain (SFL2643/YnfAComp). An MIC_90_ assay using the microtiter plate dilution method was done to examine YnfA’s ability to confer resistance against various 2-fold serial dilutions of various antimicrobials. It was seen that the MIC_90_ values of the KO mutant SFL2640 (ΔYnfA) were comparatively lesser than the 1c WT (SFL2608) and complement strain (SFL2643/YnfAComp) demonstrating increased susceptibility to the antimicrobials tested. A similar pattern of observation was seen in the drug sensitivity assay using the plate method, in which 10-fold dilutions of the *Shigella* cultures are spotted on TSA plates containing antimicrobial compounds at a particular concentration. The *ynfA* KO mutant SFL2640 (ΔYnfA) showed increased sensitivity and an altered growth pattern to the antimicrobial compounds when compared to the 1c WT (SFL2608) and complement strain (SFL2643/YnfAComp). Further analysis of the transport activity of YnfA, a fluorescence-based transport assay, was carried out with EtBr and acriflavine. It was observed that the *ynfA* KO mutant showed significantly decreased transport activity of both acriflavine and EtBr in comparison with the 1c WT (SFL2608) and complement strain (SFL2643/YnfAComp). These results demonstrated that YnfA is a functional efflux pump, involved in the transport of antimicrobials majorly cationic compounds just like other SMR transporters, and is involved in conferring resistance against them in S. flexneri.

Mutagenesis studies of the YnfA transporter were also carried out, based on previous extensive functional assessment of EmrE (39, 83–86). Nine amino acid targets of YnfA were selected based on their conserved nature to EmrE and other YnfA homologs. The nine YnfA mutants, SFL2652 (FF-LL), SFL2653 (E15A), SFL2654 (G18A), SFL2655 (WLL-QVV), SFL2656 (GGV-AAA), SFL2657 (Y60A), SFL2658 (Y63A), SFL2659 (Y67A), and SFL2660 (Y86A), along with the WT YnfA protein (SFL2643) and the pBAD_*Myc*_HisA empty vector (SFL2662) used as negative control, were subjected to MIC_90_ assay, and transport assay using acriflavine and EtBr. These assays were performed to analyze the effect of amino acid mutation on YnfA’s transport activity and its ability to confer resistance against acriflavine and EtBr. Initially, protein expression was confirmed to check if the amino acid mutation caused any expression defects. Western blot was carried out using the anti-HisA antibody and it was seen that all of the YnfA mutant proteins were expressed appropriately in the *Shigella* strains. It was observed from the functional assays, that YnfA’s functional capabilities of efflux transport and antimicrobial resistance in the presence of EtBr and acriflavine were significantly impaired in mutants: SFL2653 (E15A), SFL2654 (G18A), and SFL2657 (Y60A). Moderate to slight differences were seen in resistance profile and transport activity for the mutant SFL2658 (Y63A). Lastly, mutants SFL2652 (FF-LL), SFL2655 (WLL-QVV), SFL2656 (GGV-AAA), SFL2659 (Y67A), and SFL2660 (Y86A) showed no consequential change, displaying WT YnfA (SFL2643) transport and resistance conferment capabilities. These results can be corroborated based on the previous mutational studies done in EmrE and other SMR transporters.

Glutamic acid residue at the 14th position is highly conserved in all SMR members; in YnfA, it is present at the 15th position. Studies in EmrE have shown that mutation of the Glu14, a negatively charged membrane-embedded residue, leads to an altered substrate binding, diminished transport activity, and abolishment of resistance to toxic compounds (39, 83–85). This conserved glutamic acid in SMR transporters is understood to play a critical role in the formation of the binding domain for both substrates and protons thereby affecting the overall transport (39, 83–85). Additionally, there is a set of five amino acids in EmrE clustering around the Glu-14 are important for the proper functioning of the transporter, being involved in the recognition and translocation of substrate and protons (86). These amino acids are also involved in stabilizing drug binding by hydrophobic and electrostatic interactions (86). Mutations of amino acids in this cluster yielded the EmrE transporter to show altered affinity and binding to a substrate, along with changes in the resistance profile against toxic compounds (86). Tyrosine residues are considered important in integral membrane proteins and are involved in substrate binding. Mutation of the tyrosine residues in EmrE showed that it modifies affinity and binding to substrate, is resistance to toxic compounds, and also may diminish transport activity (89). Glycine (E18) part of the Glu-14 neighboring important amino acid cluster and tyrosine (Y60) are another set of conserved amino acids in the SMR family and are involved in transmembrane stabilization and substrate binding as shown in EmrE (86, 89). Mutational analysis of E18 and Y60 in EmrE showed that the mutants were impaired in substrate binding or affinity to substrates was lowered, resistance to toxic compounds was abolished, and transport activity was affected (86, 89). Mutations of tryptophan residue (W-63) and glycine (Gly-67) in EmrE are known to cause destabilization of the protein dimer and affected the transport activity and resistance to toxic compounds was abolished (86, 90, 91). The tyrosine at 63rd position (Y63) and amino acid triplets WLL and GGV are highly conserved in all YnfA homologs and but are not present in the EmrE protein. Mutation of these residues caused changes in the transport activity and resistance profile of the mutants, and it could be assumed that these amino acid residues play a role in YnfA’s transport mechanism and substrate binding (86, 90, 91). Phenylalanine residues are known to be involved in protein assembly and formations of alpha helices and protein-protein interactions (97, 98), but the mutation of the phenylalanine pair (FF) and also the tyrosine residues at 67th and 68th positions (Y67, Y68) did not show any changes in the resistance profile and/or the transport activity of YnfA. It can be assumed that these residues do not play critical roles in the functioning of the YnfA transporter, but additional structural and functional assessments are needed, as they can entirely validate these observations seen in the present study. Further examination of the effect of mutating these amino acid residues on the overall activity of the YnfA transporter could be done with fluorescent-tagged antibiotics. This can enable an understanding of the role of these critical amino acids in YnfA’s resistance mechanism and transport of clinically significant antibiotics utilized against *Shigella*.

Lastly, it was also seen that both YnfA and EmrE transporters contribute to the overall resistance and transport of acriflavine and EtBr in S. flexneri 1c as observed by the drastic decrease in the transport activity when both *emrE* and *ynfA* were deleted from the 1c WT (SFL 2608). However, single deletions displayed that deleting *emrE* impacted the transport more than deleting the *ynfA* transporter gene, suggesting EmrE to be a better transporter than YnfA. The presence of two SMR transporters, carrying out transport of similar antimicrobial compounds can be explained by genetic redundancy which provides an environmental adaptive advantage to bacterial pathogens and a benefit of one protein doing the job in the absence of the other (92). SMR proteins are also known to work synergistically with other resistance mechanisms such as additional efflux transporters and resistance imparting genes in bacteria (33–35). These small proteins transport an extensive variety of substrates and are believed to function as evolutionary building bricks for larger multidrug efflux proteins (33–35). A transcriptional study conducted in multidrug-resistant E. coli strains which were obtained from urinary tract infection (UTI) patients, showed a high level of expression of *ynfA* along with *tolC* in about 75% to 80% of the isolates (99). This study also indicated that the YnfA transporter works alone or in complex association with the TolC transporter to confer resistance in the MDR E. coli strains (99).

*Shigella* is known to have a good adaptive power to acquire resistance genes and with the current overuse of antimicrobials, it creates an environment that leads to a selection of resistant bacteria. This prominently causes an abundance of drug-resistant strains that becomes untreatable with existing antimicrobials. Hence, understanding the various antimicrobial resistance mechanisms employed by bacteria is essential to tackle and prevent the rise of multidrug-resistant *Shigella* strains (100). The SMR superfamily still consists of numerous uncharacterized transporter proteins which are being identified with large-scale genome sequencing projects and bioinformatic analysis of their sequences. Development of drugs or inhibitors that can be used to impair the activity of these efflux pumps can restore back bacterial susceptibility to a particular antimicrobial (101–105). Similar kind of studies in the past have led to the development of compounds that can inhibit the *tet* efflux pump which confers resistance against tetracycline (106). Advanced research is needed to understand unique SMR proteins such as YnfA, beyond the overshadowing knowledge of EmrE, which can eventually help in developing clinically useful drugs or inhibitors and can aid in combatting the growing antimicrobial resistance in *Shigella* and other bacterial pathogens.

## MATERIALS AND METHODS

### Strains and growth conditions.

Clinical strain from Bangladesh of S. flexneri serotype 1c (SFL1613/Y394) was kindly given by Nils I. A. Carlin (107). Routinely, *Shigella* strains were streaked from glycerol stocks using a sterile loop and cultured on Luria Bertani (LB) agar plates or on tryptic soy broth (TSB) agar plates with 0.01% (wt/vol) Congo red. The plates were incubated overnight at 30°C to maintain the large virulence plasmid. The overnight cultures were subsequently subcultured at a dilution of 1:100 and incubated at 37°C with shaking (180 rpm), until the desired optical density at 600 nm (OD600) was obtained. The antibiotics were added where indicated at these concentrations: 100 μg/mL ampicillin, 50 μg/mL kanamycin, 25 μg/mL chloramphenicol. All bacterial strains and plasmids used in this study are recorded in Tables S2 and S3.

### Generating the *ynfA* and *emrE* gene KO mutants.

The lambda red double homologous recombination technique was utilized to create the gene knockouts (108, 109). The helper plasmid pKD46, showing inducible expression of the lambda red genes (gam, beta, and exo), along with pKD3 (chloramphenicol resistance) and pKD4 (kanamycin resistance) plasmids were used for generating knockouts via a PCR based approach. In this method, the resistance genes of pKD3 and pKD4 plasmids were amplified using primer pairs having 80 bp overhangs of homologous regions upstream and downstream of the target gene to be deleted. Positive gene knockout mutants were screened on LBA plates containing appropriate antibiotics, followed by colony PCR and Sanger sequencing. Primers amplifying the *apy* and *virG* genes were used to confirm the presence of an intact *Shigella* virulence plasmid in the KO mutant strain. All PCR primers used in this study are recorded in Table S4.

### Gene complementation, site directed mutagenesis, and protein expression.

Gene complementation and site-directed mutants were made by GenScript USA and cloned into the expression pBAD_*Myc*_HisA vector using NcoI and HindIII, along with BamHI and ApaI sites. The positive clones were selected using erythromycin and ampicillin as the pBAD_*Myc*_HisA vector has resistance genes for both the antibiotics. Furthermore, the potential clones were also confirmed via Sanger sequencing using the pBAD universal primers.

For protein expression, *Shigella* strains were grown in LB broth medium having 50 μg/mL erythromycin and 100 μg/mL ampicillin to maintain the pBAD_*Myc*_HisA_YfiB vector, at 37°C. Once log phase cultures are obtained (OD600 = 0.5 to 0.6), 1 mL of culture was pelleted down and resuspended in the β-mercaptoethanol protein loading dye. Arabinose induction was not carried out, as there is always a basal level of expression of the cloned gene of interest even without arabinose and overexpression of proteins is not possible in *Shigella* strains (110). Protein suspensions were boiled for 5 min before loading 10 μL of the samples into the wells of two SDS gels. Equal total protein loading was confirmed using Coomassie blue staining of one gel and the other gel was used for Western blot transfer to check for protein expression via the anti-HisA antibody.

### MIC analysis.

MIC analysis was conducted using the microtiter broth dilution method using 96-well sterile microtiter plates (111). Using a multipipettor, 100 μL of sterile TSB, was added to all the wells of the plate. Then, 100 μL of an appropriate antimicrobial compound, at double the highest concentration, was added into the wells in column 1 and subsequent serial dilutions of the antimicrobial agent were made in the following columns. Overnight cultures of *Shigella* strains being tested were diluted 1:50 in 10 mL of fresh LB broth and grown to mid-log phase (OD600 = 0.5 to 0.6), as the appropriate inoculum size for standard MIC determination is 10^4^ to 10^5^ CFU/mL. A total of 5 μL of bacterial cultures were dispensed in each well containing different concentrations of the antimicrobial compound and also in the bacterial growth control well containing only TSB media. One of the wells was also designated as the media control, which contained uninoculated TSB media. The plates were incubated at 37°C for 16 h to 18 h and the MIC was determined as the lowermost concentration of antimicrobial compound at which 90% growth inhibition was seen. Growth absorbance was measured at 600 nm wavelength (OD600), using a microplate reader.

### Growth curve analysis.

Overnight bacterial cultures were diluted 1:50 in 25 mL TSB. This dilution was designated time zero, the cultures were incubated at 37°C in a shaking incubator (180 rpm). At 60-min intervals, 1 mL of culture was transferred to a microcuvette and the optical density of the suspension was measured at 600 (OD600) on a visible light spectrophotometer against an TSB blank. Graphs of the optical density versus time were plotted to generate growth curves.

### Drug sensitivity assay.

Drug sensitivity assays were carried out using a plate-dilution method (112). Overnight cultures of *Shigella* strains being tested were diluted 1:50 in 10 mL of fresh LB broth and grown to mid-log phase (OD600 = 0.5 to 0.6). A series of 10-fold dilutions of each *Shigella* strain was prepared and 5 μL of 10-fold dilutions of each strain was spotted on the TSB agar plates containing different antimicrobial compounds. Colony growth patterns were observed after overnight incubation of these plates at 37°C. The antimicrobial compounds tested included ethidium bromide (100 μg/mL), acriflavine (25 μg/mL), methyl viologen (200 μg/mL), and crystal violet (15 μg/mL). A TSA plate not containing any drug was used as a control to assess the growth of all the 10-fold dilutions for each strain.

### Transport assays with ethidium bromide.

Accumulation of EtBr in *Shigella* was determined by diluting overnight cultures of *Shigella* strains by 1:100 in 10 mL of fresh LB broth and grown to mid-log phase (OD600 = 0.6). Cells were then centrifuged for 5 min at 13,000 rpm and the resulting pellet was washed with 1X PBS. The OD of this suspension was then adjusted to 0.3 in 1X PBS to obtain a concentration of 3.6 × 10^8^ cells/mL. Additionally, glucose was added to this cellular suspension in a final concentration of 0.4% vol/vol. Then, 180 μL of this cell suspension was dispensed to the wells of a microtiter plate, to which 20 μL of EtBr was added at a final concentration of 2 μg/mL. Four technical repeats of each strain were analyzed in each column along with a PBS blank well and only-EtBr solution well which was taken as controls. The plate was then transferred to a microplate reader (Tecan infinite M1000 pro), incubated at 37°C and fluorescence was read from the top of the wells using excitation and emission filters at 518 and 605 nm, respectively. Readings were taken for 60 min, at a default gain multiplier, with five flashes/well and a 75-s delay between cycles. Arbitrary units were recorded for fluorescence emitted at the end of each cycle (76, 78).

Ethidium bromide efflux in *Shigella* is determined by diluting overnight cultures of *Shigella* strains by 1:100 in 10 mL of fresh LB broth and grown to mid-log phase (OD600 = 0.6). Cells were then centrifuged for 5 min at 13,000 rpm and the resulting pellet was washed with 1X PBS. To this suspension, EtBr was added at a final concentration of 2 μg/mL, and this suspension was incubated at 25°C without the presence of glucose for 60 min, which caused a maximum level of accumulation of EtBr in the cells. After 60 min of incubation, 100 μL of this suspension was added to the wells of a microtiter plate. Four technical repeats of each strain were analyzed in each column along with a PBS blank well and only-EtBr solution well which was taken as controls. The plate was then transferred to a microplate reader (Tecan infinite M1000 pro). Glucose was added to this suspension in a final concentration of 0.4% vol/vol to start the efflux process and incubated at 37°C. Fluorescence was read from the top of the wells using excitation and emission filters at 518 and 605 nm, respectively. Readings were taken for 60 min, at a default gain multiplier, with five flashes/well and a 75-s delay between cycles. Arbitrary units were recorded for fluorescence emitted at the end of each cycle (76, 78).

### Transport assays with acriflavine.

Overnight cultures of *Shigella* strains were diluted 1:100 in 10 mL of fresh LB broth and grown to mid-log phase (OD600 = 0.6). Cells were then centrifuged for 5 min at 13,000 rpm and the resulting pellet was washed with 20 mM HEPES (pH 7.0). The OD of this cellular suspension was then adjusted to 0.3 in HEPES to obtain a concentration of 3.6 × 10^8^ cells/mL. Additionally, glucose was added to this cellular suspension in a final concentration of 0.4% vol/vol. Then, 180 μL of this suspension was dispensed to the wells of a microtiter plate, to which 20 μL of Acriflavine was added at a final concentration of 5 μM. Four technical repeats of each strain were analyzed in each column along with a PBS blank well and only-acriflavine solution well which was taken as controls. The plate was then transferred to a microplate reader (Tecan infinite M1000 pro), incubated at 37°C and fluorescence was read from the top of the wells using excitation and emission filters at 450 and 510 nm, respectively. Readings were taken for 60 min, at a default gain multiplier, with five flashes/well and a 75-s delay between cycles. Arbitrary units were recorded for fluorescence emitted at the end of each cycle (113, 114).

Acriflavine efflux in *Shigella* is determined by diluting overnight cultures of *Shigella* strains by 1:100 in 10 mL of fresh LB broth and grown to mid-log phase (OD600 = 0.6). Cells were then centrifuged for 5 min at 13,000 rpm and the resulting pellet was washed with 20 mM HEPES (pH 7.0). To this cell suspension, acriflavine was added at a final concentration of 5 mM and this suspension was incubated at 25°C without the presence of glucose for 60 min, which caused a maximum level of accumulation of acriflavine in the cells. After 60 min of incubation, 100 μL of this suspension was added to the wells of a microtiter plate. Four technical repeats of each strain were analyzed in each column along with a PBS blank well and only-acriflavine solution well which was taken as controls. The plate was then transferred to a microplate reader (Tecan infinite M1000 pro), glucose was added to this suspension in a final concentration of 0.4% vol/vol to start the efflux process and incubated at 37°C. Fluorescence was read from the top of the wells using excitation and emission filters at 450 and 510 nm respectively. Readings were taken for 60 min, at a default gain multiplier, with five flashes/well and a 75-s delay between cycles. Arbitrary units were recorded for fluorescence emitted at the end of each cycle (113, 114).

### Statistical analysis.

Statistical significance was evaluated in every experiment based on a minimum of three trials and was performed using the GraphPad Prism’s unpaired Student’s *t* test with a required cut-off for significance being *P* < 0.05.

### Bioinformatic analysis of YnfA protein.

The five-step *in silico* approach for analyzing hypothetical proteins was applied initially for the YnfA protein sequence (115). The transmembrane helices were predicted using the TMHMM (55) and TMpred (56) bioinformatic tools. The similarity in between the protein sequences and identification of amino acid residues which are conserved was performed using NCBI-protein BLAST (116) and Clustal-Omega sequence alignment tool (57). Using the Clustal-Omega alignment, sequence conservation at each amino acid position in a protein was also analyzed using the WebLogo tool (59). Weblogo generates a pile of amino acid symbols, indicating the most conserved residues at each position via the altitude of the symbols (59). I-TASSER (Iterative Threading ASSEmbly Refinement) tool (62, 63, 117) was employed to work out 3D structure of YnfA protein. The resulting predicted structure of YnfA was also validated using the AlphaFold protein structure database which has high accuracy of structure prediction (64).

Phylogenetic analysis of YnfA with its homologs and other members belonging to the SMR family was carried out using the MEGA software (58). First a multiple-sequence alignment was performed and the maximum composite likelihood method was utilized to calculate the evolutionary distances between the protein sequences to construct illustrative phylogenetic trees (118). UniProt accession IDs of all SMR family members and YnfA homolog protein sequences used in this study are listed in Table S5.

### Data availability.

Additional data is provided in the supplementary file.
