# Supplementary material for: Functional Role of YnfA, an Efflux Transporter in Resistance to Antimicrobial Agents in Shigella flexneri
Source: Antimicrob Agents Chemother. 2022 Jun 21;66(7):e00293-22. doi: 10.1128/aac.00293-22 (PMC9295541; doi:10.1128/aac.00293-22)
Supplement: Supplemental file 1 — Fig. S1 to S7 and Tables S1 to S5. Download aac.00293-22-s0001.pdf, PDF file, 0.9 MB [file aac.00293-22-s0001.pdf]

Supplementary materials

Functional role of YnfA, an Efflux Transporter in Resistance to Antimicrobial Agents in *Shigella flexneri*

Tanuka Sen (1), Naresh K Verma (1)*

Authors affiliation

Division of Biomedical Science and Biochemistry, Research School of Biology, The Australian National University, ACT, Canberra, Australia.

Corresponding author: Naresh K. Verma.

This file includes Supplementary Figures S1- S7 and Supplementary Tables S1- S5.

A

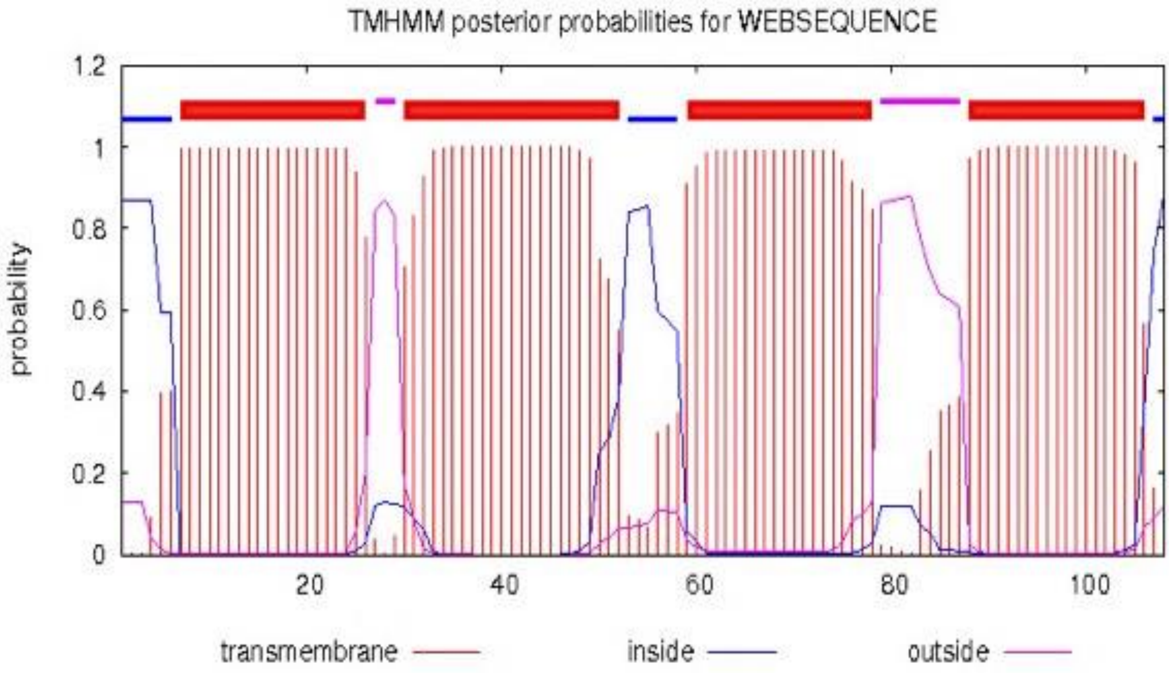

B

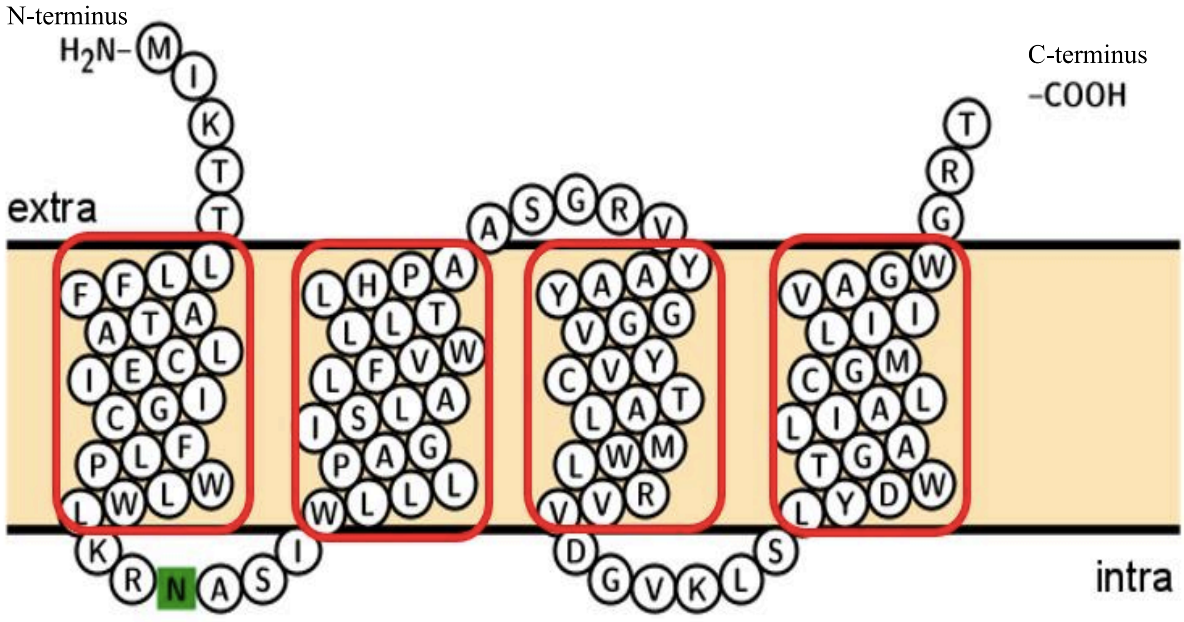

Supplementary Figure 1. Transmembrane alpha-helices segments of YnfA. A) TMHMM and TMpred programs predicted four transmembrane helices. The plot shows transmembrane regions in red, an inside-outside and outside-inside configuration in blue and purple respectively. **B)** The secondary structure of YnfA membrane transporter from *S. flexneri* 1c, as determined by hydropathy analysis and also based on EmrE's secondary structure. Extra- extracellular, intra- intracellular.

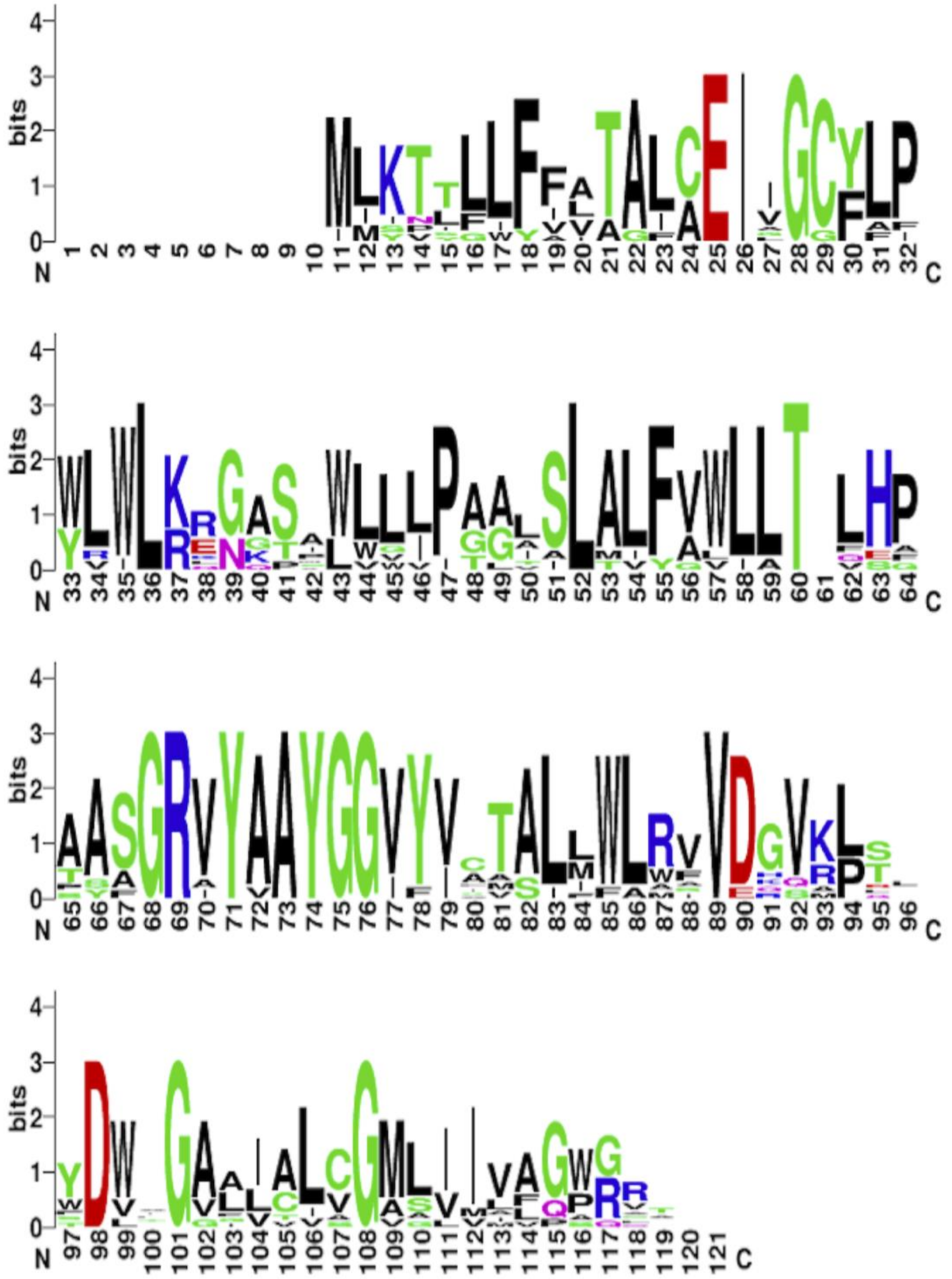

Supplementary Figure 2. Weblogo representation of the amino acid conservation in YnfA protein. The tallness of the amino acid code at each position reveals the relative occurrence of the amino acid at that position and the total alititude of the pile denotes the amount of conservation. (measured in bits). The consensus sequence was obtained from a protein sequence alignment of YnfA protein homologs from various Gram-negative bacteria. UniProt accession IDs of the YnfA protein homologs, used for the phylogenetic analysis are listed in **Supplementary Table S5.**

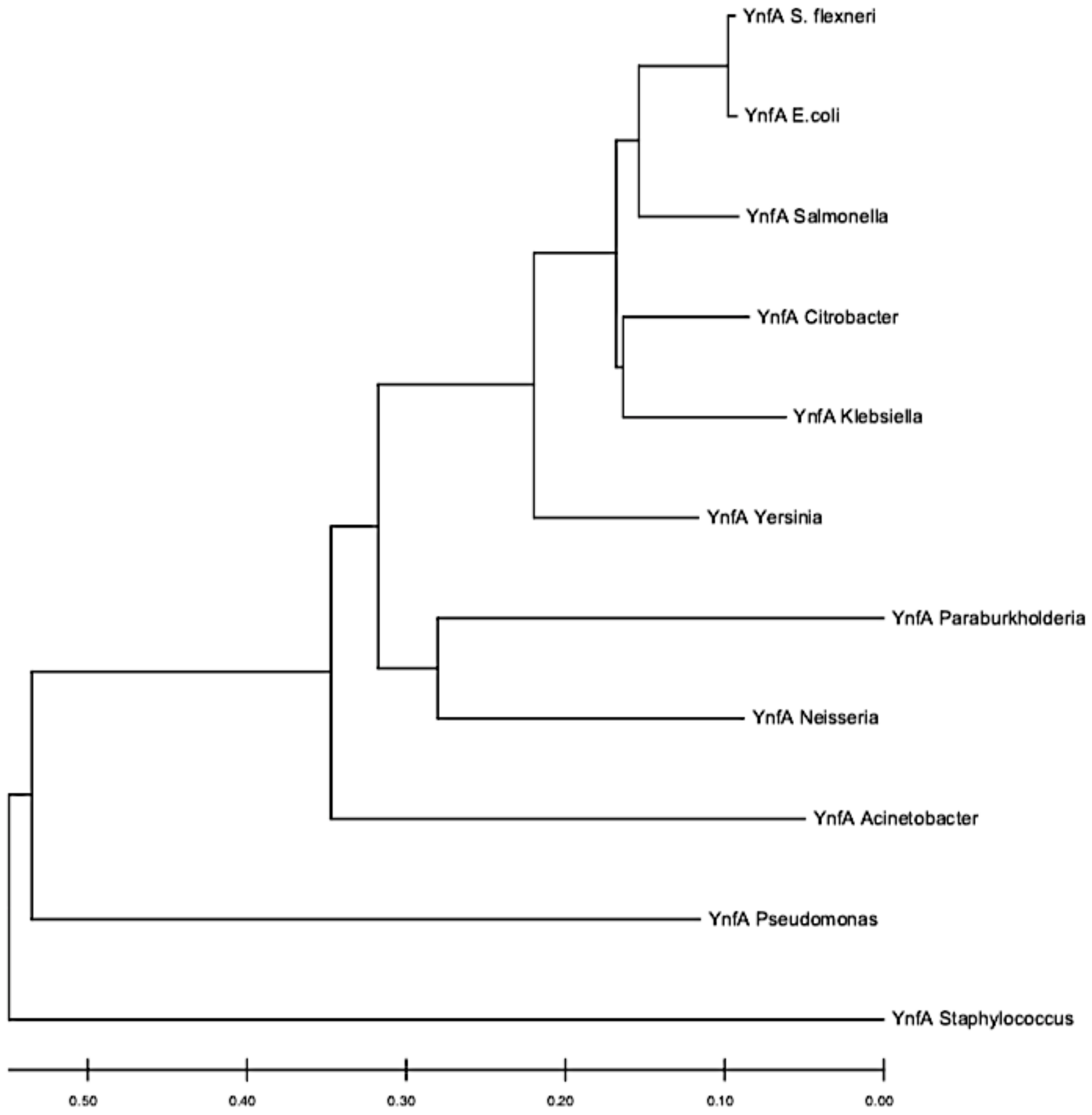

Supplementary Figure 3. Phylogenetic tree displaying the evolutionary distance between the YnfA protein homologs from several Gram-negative pathogens. Clustal-Omega alignment of the homologs was utilized to create this phylogenetic tree, using the MEGA software and computed by the Maximum Composite Likelihood method. UniProt accession IDs of the YnfA protein homologs, used for the phylogenetic analysis are listed in **Supplementary Table S5.**

A

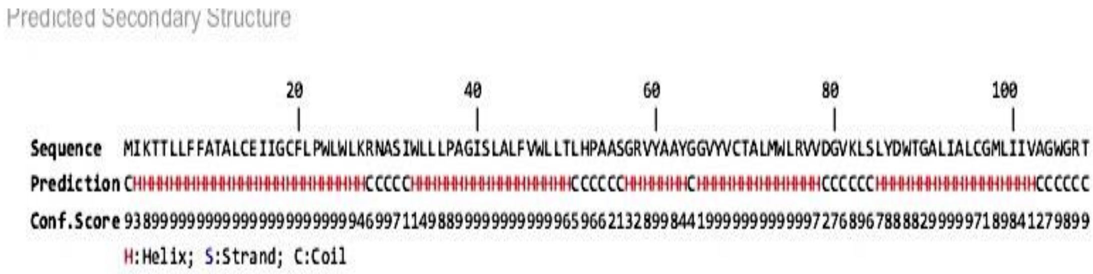

B

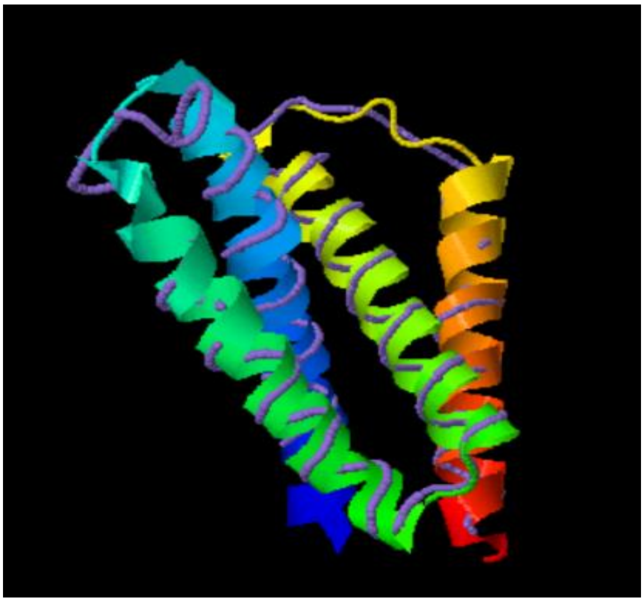

Protein used as a template to thread: EmrE
PDB ID: 3b61
Identity 1*: 0.23
Identity 2*: 0.24
Coverage*: 0.95
Normalized Z-score*: 2.15

Supplementary Figure 4. The 3D structure of YnfA protein, predicted using the I-TASSER tool. A) Predicted secondary structure of YnfA, showing the query protein sequence with residue numbers. Each residue is assigned to either an (H) alpha helix, (T) beta-turn or (C) random coil and given a score. The score ranges from 0 to 9, with 0 indicating buried residues and 9 exposed residues**. B)** Diagrammatic representation of threading of YnfA protein sequence against the template of EmrE structure. YnfA query protein sequence is shown in cartoon, while the structural analog (EmrE) is displayed using backbone trace. *Ident1 is the percentage sequence identity of the templates in the threading aligned region with the query sequence. *Ident2 is the percentage sequence identity of the whole template chains with query sequence. * Cov represents the coverage of the threading alignment and is equal to the number of aligned residues divided by the length of query protein. *Norm. Z-score is the normalized Z-score of the threading alignments, where a Z-score >1 means a good alignment.

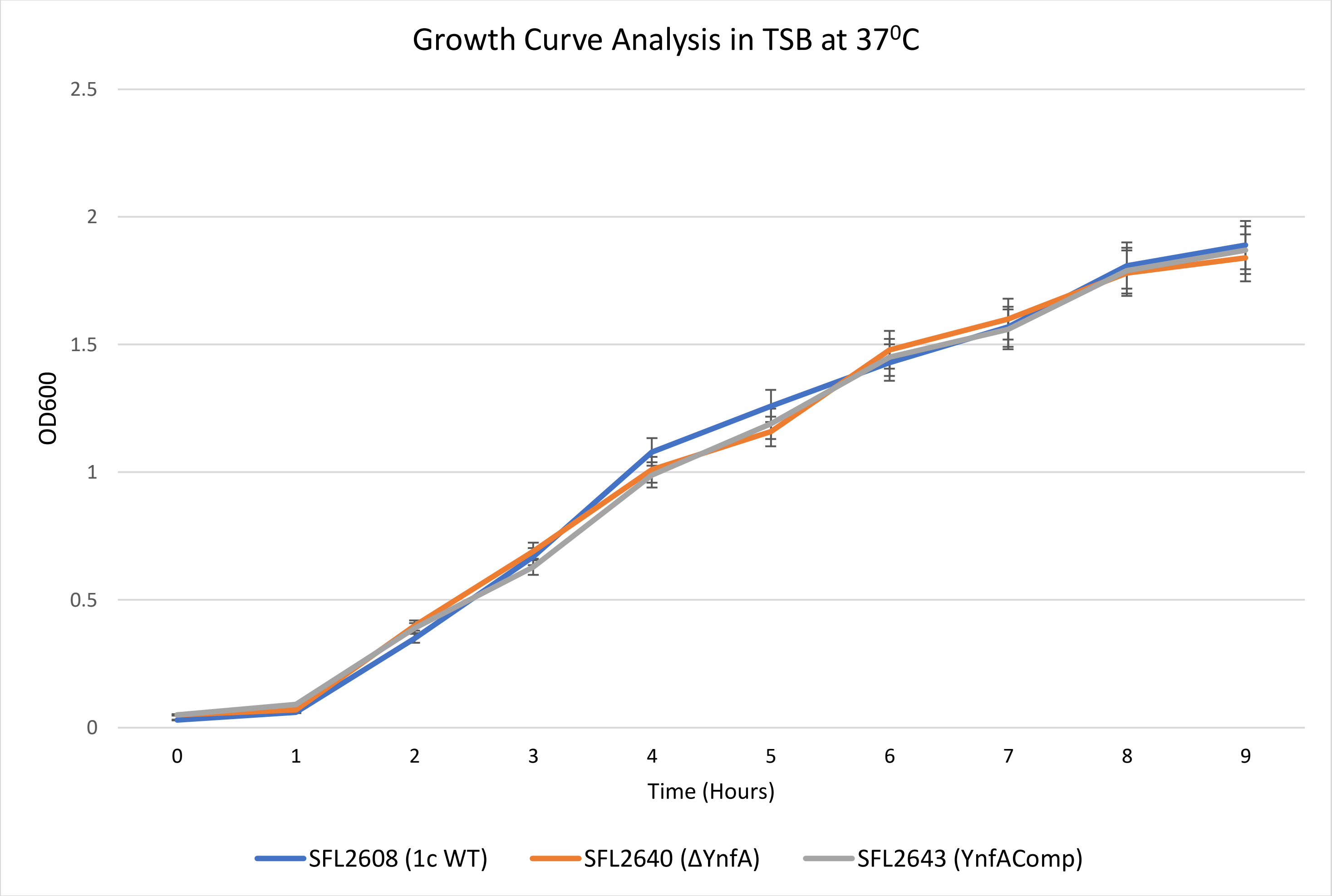

Supplementary Figure 5. Growth curves for the wild-type, KO mutant and complement strains in TSB media at 37^{0}C. The OD600 for the wildtype strain SFL2608 (1c WT) ynfA KO mutant SFL2640 (ΔYnfA) and the ynfA complement strain SFL2643 (YnfAComp) was plotted against time. The results were based on the mean average of three independent repeats and the error bars represent the standard deviation.

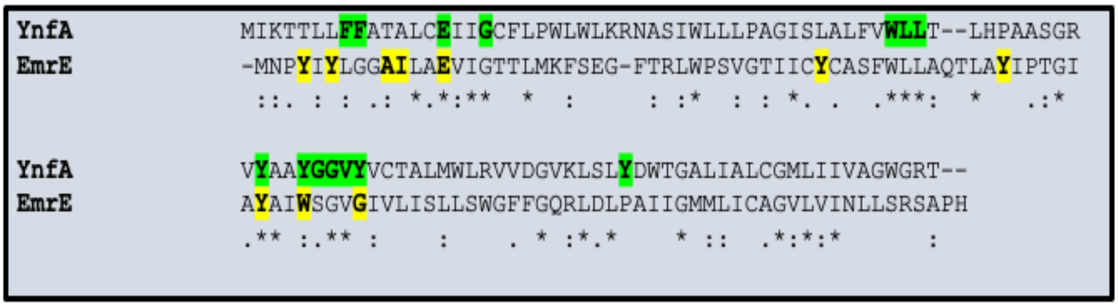

Supplementary Figure 6. Multiple sequence alignment of *E. coli* EmrE protein and YnfA protein from *S. flexneri* 1c. The critical amino acid residues known to be critical for the proper functioning of EmrE are highlighted in Yellow and the amino acid residues selected as the target for mutagenesis studies in YnfA are highlighted in green. Symbols denoting- **:** strongly similar amino acid residue; **.** weakly similar amino acid residue; ***** conserved amino acid residue.

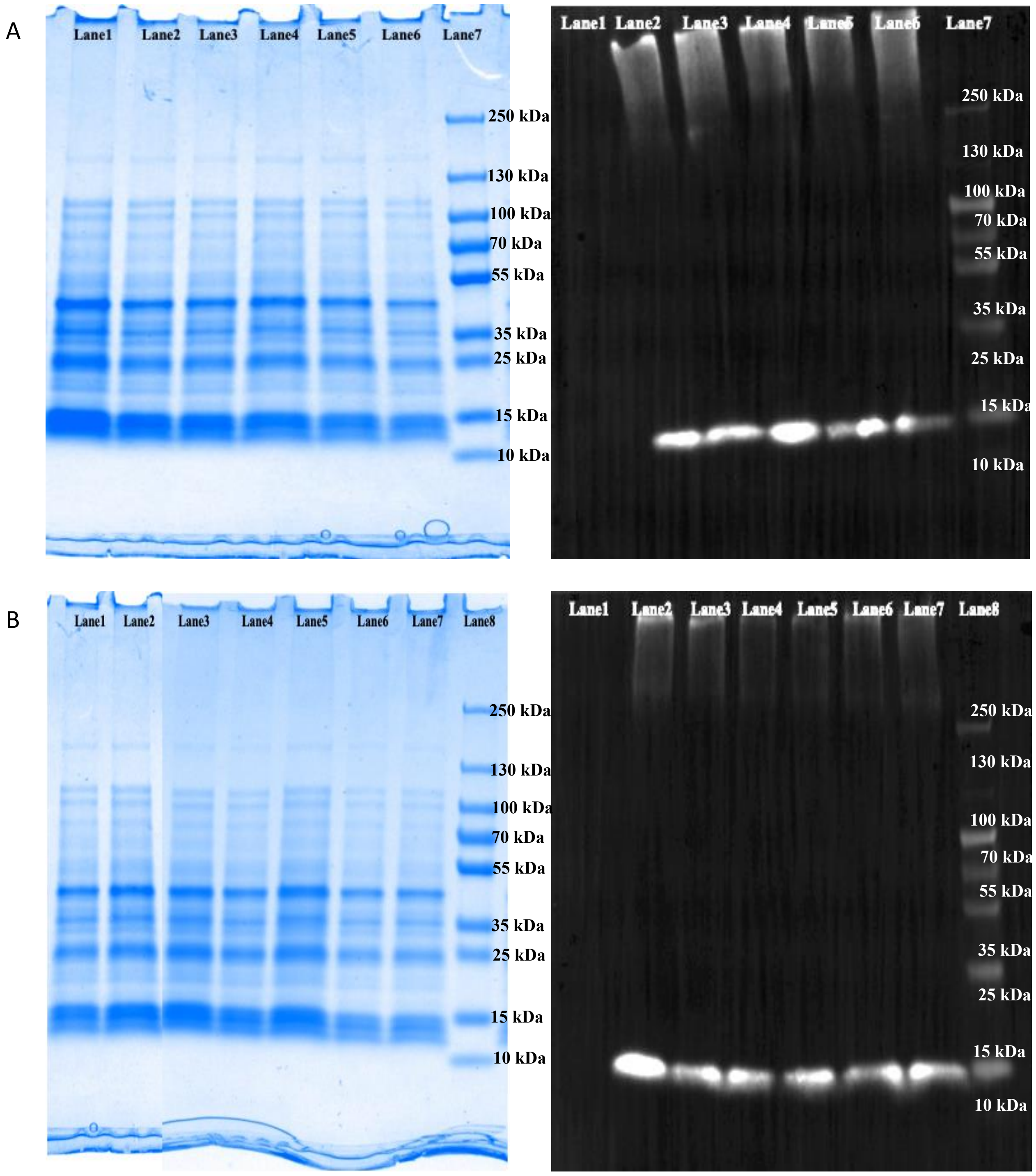

Supplementary Figure 7. Confirming protein expression of the WT YnfA and the site-directed YnfA mutants. Displayed here are the SDS gels stained with Coomassie blue for validating equal total protein loading for each sample, followed by the Western transfer and blotting of the loaded SDS gel using the anti-HisA antibody to check for correct protein

expression. YnfA is known to be an 11.9 kDa protein, wildtype YnfA protein sample, and site-directed mutants of YnfA, all show a band at the same location below the 15 kDa band of the protein ladder. **A)** Lane1- pBAD_*Myc*_HisA empty vector (SFL2662), Lane2- WT YnfA protein (SFL2643/ YnfAComp strain), Lane3- SFL2652 (FF-LL), Lane4- SFL2653 (E15A), Lane5- SFL2654 (G18A) and Lane6- SFL2655 (WLL-QVV). **B)** Lane1- pBAD_*Myc*_HisA empty vector (SFL2662), Lane2- WT YnfA protein (SFL2643/ YnfAComp strain), Lane3- SFL2656 (GGV-AAA), Lane4- SFL2657 (Y60A), Lane5- SFL2658 (Y63A), Lane6- SFL2659 (Y67A), and Lane7- SFL2660 (Y86A).

Table S1-List of amino acid targets chosen for mutagenesis studies of YnfA. The table includes the target amino acid positions, which amino acid it has been mutated along with its employed criteria and the strain nomenclature used in this study.

Target amino acid and position	Mutated amino acid	Employed Criteria	Mutant nomenclature used in this study
FF-(8, 9)	LL	Phenylalanine can be substituted by other aromatic or hydrophobic amino acids like leucine	SFL2652 (FF-LL)
E-15	A	Glutamic acid (E) can be substituted by other small amino acids like alanine (A)	SFL2653 (E15A)
G-18	A	Glycine (G) can be substituted by other small amino acids like alanine (A)	SFL2654 (G18A)
WLL- (47, 48, 49)	QVV	Tryptophan (W) Can be replaced by other small, aromatic amino acids like glutamine (Q)<br>Leucine (L) can be substituted by other small, hydrophobic, aliphatic amino acids like valine (V)	SFL2655 (WLL-QVV)
GGV- (64, 65, 66)	AAA	Glycine (G) and Valine (V) can be substituted by other small amino acids like alanine (A)	SFL2656 (GGV-AAA)
Y-60	S	Tyrosine (Y) can be substituted by other aromatic amino acids like serine (S)	SFL2657 (Y60A)
Y-63	S	Tyrosine (Y) can be substituted by other aromatic amino acids like serine (S)	SFL2658 (Y63A)
Y-67	S	Tyrosine (Y) can be substituted by other aromatic amino acids like serine (S)	SFL2659 (Y67A)
Y-86	S	Tyrosine (Y) can be substituted by other aromatic amino acids like serine (S)	SFL2660 (Y86A)

Table S2- Bacterial strains used in this study.

Name	Description	Antibiotic Resistance
Shigella flexneri		
SFL1613/Y394	*S. flexneri* 1c strain[104]	-
SFL2608 (1c WT)	SFL1613/Y394 with pKD46 helper plasmid. Wildtype used for this study.	Ampicillin
SFL2640 (ΔYnfA)	SFL2608 with *ynfA* gene knocked out	Chloramphenicol
SFL2643(YnfAComp)	SFL2640 with *ynfA* gene complemented, cloned into pBAD_*Myc*_HisA vector	Chloramphenicol, Ampicillin, Erythromycin
SFL2652	SFL2640 with site directed mutant-(FF-LL) cloned into pBAD_*Myc*_HisA vector.	Chloramphenicol, Ampicillin, Erythromycin
SFL2653	SFL2640 with site directed mutant-(E15A) cloned into pBAD_*Myc*_HisA vector.	Chloramphenicol, Ampicillin, Erythromycin
SFL2654	SFL2640 with site directed mutant-(G18A) cloned into pBAD_*Myc*_HisA vector.	Chloramphenicol, Ampicillin, Erythromycin
SFL2655	SFL2640 with site directed mutant-(WLL-QVV) cloned into pBAD_*Myc*_HisA vector.	Chloramphenicol, Ampicillin, Erythromycin
SFL2656	SFL2640 with site directed mutant-(GGV-AAA) cloned into pBAD_*Myc*_HisA vector.	Chloramphenicol, Ampicillin, Erythromycin
SFL2657	SFL2640 with site directed mutant-(Y60A) cloned into pBAD_*Myc*_HisA vector.	Chloramphenicol, Ampicillin, Erythromycin
SFL2658	SFL2640 with site directed mutant-(Y63A) cloned into pBAD_*Myc*_HisA vector.	Chloramphenicol, Ampicillin, Erythromycin
SFL2659	SFL2640 with site directed mutant-(Y67A) cloned into pBAD_*Myc*_HisA vector.	Chloramphenicol, Ampicillin, Erythromycin
SFL2660	SFL2640 with site directed mutant-(Y86A) cloned into pBAD_*Myc*_HisA vector.	Chloramphenicol, Ampicillin, Erythromycin
SFL2662	SFL 2640 with empty pBAD_*Myc*_HIsA vector	Chloramphenicol, Ampicillin, Erythromycin
SFL2644	SFL2640 (ΔYnfA) with *emrE* gene knocked out	Kanamycin, Chloramphenicol
SFL2661	SFL2608 with *emrE* gene knocked out	Kanamycin

Table S3- Bacterial plasmids used in this study.

**Plasmids**		
pKD46	Helper plasmid expressing the lambda red genes (*gam, beta*, and *exo*)	Ampicillin
pKD3	Used for template generation for homologous recombination.	Chloramphenicol
pBAD_*Myc*_HisA	Vector used for cloning *ynfA* gene, used as control empty vector.	Ampicillin, Erythromycin
pBAD_*Myc*_HisA_YnfA	pBAD_*Myc*_HisA with *ynfA* gene cloned at the *Nco*I and *Hind*III.	Ampicillin, Erythromycin
pBAD_*Myc*_HisA_M1	pBAD_*Myc*_HisA with mutant YnfA (FF-LL)	Ampicillin, Erythromycin
pBAD_*Myc*_HisA_M2	pBAD_*Myc*_HisA with mutant YnfA (E15A)	Ampicillin, Erythromycin
pBAD_*Myc*_HisA_M3	pBAD_*Myc*_HisA with mutant YnfA (G18A)	Ampicillin, Erythromycin
pBAD_*Myc*_HisA_M4	pBAD_*Myc*_HisA with mutant YnfA (WLL-QVV)	Ampicillin, Erythromycin
pBAD_*Myc*_HisA_M5	pBAD_*Myc*_HisA with mutant YnfA (GGV-AAA)	Ampicillin, Erythromycin
pBAD_*Myc*_HisA_M6	pBAD_*Myc*_HisA with mutant YnfA (Y60A)	Ampicillin, Erythromycin
pBAD_*Myc*_HisA_M7	pBAD_*Myc*_HisA with mutant YnfA (Y63A)	Ampicillin, Erythromycin
pBAD_*Myc*_HisA_M8	pBAD_*Myc*_HisA with mutant YnfA (Y67A)	Ampicillin, Erythromycin
pBAD_*Myc*_HisA_M9	pBAD_*Myc*_HisA with mutant YnfA (Y86A)	Ampicillin, Erythromycin

Table S4- Primers used in this study.

**Primer Name**	**Role**	**Sequence (5'-3')**
*ynfA*_For	Primer for amplifying the CM gene of pKD3, containing the 80bp overhangs, homologous to the upstream of the *ynfA* gene	CATCCCCAAGGCCCGTAA TACCGTCCTCAGTGGTGATT TTTAATGTGACGAAATTACG CCCCGGACAGGTAACAAA AACTtacacgtcttgagcgattgt
*ynfA*_Rev	Primer for amplifying the CM gene of pKD3, containing the 80bp overhangs, homologous to the downstream of the *ynfA* gene	GCAAAACGTACCAGGAGG ATGTTTATATTGATGATATTAT GTCGCCCTATAACTATACAT GATGTCAATAAGTGACAA AGggtccatatgaatatcctcc
*ynfA*_Test_For	Confirming deletion of *ynfA* gene	CAATCAGTTGTTTGCAGTCCC
*ynfA*_Test_Rev	Confirming deletion of *ynfA* gene	CGATTCGTTTGCTGAGAGTG
*emrE*_For	Primer for amplifying the Kan gene of pKD4, containing the 80bp overhangs, homologous to the upstream of the *emrE* gene	ATCATAATTATAAAGGTGCCCCGAACA TTAGCCATAATATGCACAAATCAGATT CCTGTTATCTCAAGGAGAATTATTTTtac acgtcttgagcgattgt
*emrE*_Rev	Primer for amplifying the CM gene of pKD4, containing the 80bp overhangs, homologous to the downstream of the *emrE* gene	AGCATATTCTTTCCTGTTCATACTGGAG AGAATTGTACTACAGTTTGAACACAAT CACCGTTTTCATCCTGGTGTATGTggtcca tatgaatatcctcc
*emrE*_Test_For	Confirming deletion of *emrE* gene	TATAAAGGTGCCCCGAACAT
*emrE*_Test_Rev	Confirming deletion of *emrE* gene	CCAGGATGAAAACGGTGAGT
CM_Test	Confirming deletion of *ynfA* gene and presence of chloramphenicol gene	CAACGGTGGTATATCCAGTG
Apy_For	Checking presence of *Shigella*'s Virulence plasmid	CATAATCAAGAGACAAAACGATA
Apy_Rev	Checking presence of *Shigella*'s Virulence plasmid	CCAGCCTTTCCAGTAATCCC

Table S4- Primers used in this study.

VirG_For	Checking presence of *Shigella*'s Virulence plasmid	CGGGTACTCAAGAACTTCAAT
VirG_Rev	Checking presence of *Shigella*'s Virulence plasmid	TTCCGCCAAAATGAGAGTTCC
pBAD_Univ_For	Primer to check for correct cloning in the pBAD_Myc_HisA vector	ATGCCATAGCATTTTTATCC
pBAD_Uni_Rev	Primer to check for correct cloning in the pBAD_Myc_HisA vector	GATTTAATCTGTATCAGG

Table S5- UniProt accession IDs of protein sequences used in this study.

Protein Name	UniProt Accession ID
YnfA	Q83L10
YvdS	Q65J62
YkkC	Q65KV1
SugE	P69937
YvdR	I2C1Z0
Cj1173	Q0P983
YdgF	A0A0F7J891
YdgE	P69210
EbrA	P0CW80
QacG	O87866
ebr	P14319
YvaE	A0A2P0HGJ0
EmrE	P23895
QacE	P0AGC9
QacE2	A0A2Z5WQM8
QacF	Q9X2N9
YnfA_*S. flexneri*	Q83L10
YnfA_*E. coli*	P76169
YnfA_*Salmonella*	Q5PHH0
YnfA_*Citrobacter*	A0A336QQP9
YnfA_*Klebsiella*	W9BP83
YnfA_*Yersinia*	A0A0E1NMC1
YnfA_*Paraburkholderia*	A0A1N7SA05
YnfA_*Neisseria*	A0A448UCB0
YnfA_*Acinetobacter*	A0A0D5YGX4
YnfA_*Pseudomonas*	A0A232B3A0
YnfA_*Staphylococcus*	A0A0M0QSA6
